# Differential plant cell responses to *Acidovorax citrulli* T3SS and T6SS reveal an effective strategy for controlling plant-associated pathogens

**DOI:** 10.1128/mbio.00459-23

**Published:** 2023-06-08

**Authors:** Yumin Kan, Yanjie Zhang, Wenhui Lin, Tao Dong

**Affiliations:** 1 State Key Laboratory of Microbial Metabolism, Joint International Research Laboratory of Metabolic and Developmental Sciences, School of Life Sciences and Biotechnology, Shanghai Jiao Tong University, Shanghai, China; 2 Department of Immunology and Microbiology, School of Life Sciences, Southern University of Science and Technology, Shenzhen, Guangdong, China; University of Nebraska-Lincoln, Lincoln, Nebraska, USA

**Keywords:** *Acidovorax citrulli*, T6SS, interspecies competition, RNA-Seq, biocontrol

## Abstract

*Acidovorax citrulli* is a gram-negative plant pathogen that employs the type Ⅲ secretion system (T3SS) to infect cucurbit crops and cause bacterial fruit blotch. This bacterium also possesses an active type Ⅵ secretion system (T6SS) with strong antibacterial and antifungal activities. However, how plant cells respond to these two secretion systems and whether there is any cross talk between T3SS and T6SS during infection remain unknown. Here, we employ transcriptomic analysis to compare cellular responses to the T3SS and the T6SS during *in planta* infection and report distinctive effects on multiple pathways. The T3SS-mediated differentially expressed genes were enriched in the pathways of phenylpropanoid biosynthesis, plant-pathogen interaction, MAPK signaling pathway, and glutathione metabolism, while the T6SS uniquely affected genes were related to photosynthesis. The T6SS does not contribute to the *in planta* virulence of *A. citrulli* but is critical for the survival of the bacterium when mixed with watermelon phyllosphere bacteria. In addition, T3SS-mediated virulence is independent of the T6SS, and the inactivation of the T3SS does not affect the T6SS-mediated competition against a diverse set of bacterial pathogens that commonly contaminate edible plants or directly infect plants. A T6SS-active T3SS-null mutant (Ac_av_) could inhibit the growth of *Xanthomonas oryzae* pv. *oryzae* significantly both *in vitro* and *in vivo* and also reduce symptoms of rice bacterial blight. In conclusion, our data demonstrate the T6SS in *A. citrulli* is nonpathogenic to the plant host and can be harnessed as a pathogen killer against plant-associated bacteria.

**IMPORTANCE:**

Chemical pesticides are widely used to protect crops from various pathogens. Still, their extensive use has led to severe consequences, including drug resistance and environmental contamination. Here, we show that an engineered T6SS-active, but avirulent mutant of *Acidovorax citrulli* has strong inhibition capabilities against several pathogenic bacteria, demonstrating an effective strategy that is an alternative to chemical pesticides for sustainable agricultural practices.

## INTRODUCTION

The global demand for crop production is growing at an alarming rate as a result of population growth (1, 2). However, crop disease caused by various plant pathogens is a primary threat to global food production (3). Additionally, the extensive use of chemical pesticides has led to resistance and contamination, which poses an urgent concern to environmentally friendly agricultural management (4 - 7). Plant growth-promoting rhizobacteria have been identified as a potential solution for biocontrol applications (8). In addition, a few species of bacteria and fungi, including *Bacillus subtilis*, *Pseudomonas protegens*, and *Trichoderma harzianum*, exhibit biocontrol potential against bacterial or fungal pathogens (9 - 13). However, there are significant challenges in the large-scale application of these biocontrol agents due to their low efficiency or limited availability (14). Therefore, effective strategies tailored to specific environments for increasing crop yields and protecting against pathogens are in an urgent need.

Phytobacteria live in a complex environment that exposes them to diverse biotic and abiotic stresses. Gram-negative bacteria have evolved a few specialized protein secretion systems to adapt to environmental stresses, cope with the innate immunity of the plant host, and compete with the neighboring microbes (15, 16). The type VI secretion system (T6SS) is one of these secretion apparatuses consisting of at least 13 conserved subunits, which assemble a membrane complex (TssJLM), a baseplate (TssK-TssEFG), and a double tubular structure consisting of an outer VipA/B (TssB/C) sheath and an inner needle-like Hcp tube (17 - 20). Bacteria employ the T6SS to deliver toxic proteins into the neighboring cells, including prokaryotes and eukaryotes (17, 21 - 25). In addition, the T6SS is involved in multiple cellular processes such as metal acquisition, horizontal gene transfer, pathogenicity in the host plants, and activation of the pyrin inflammasome to trigger inflammation in the host (26 - 29). Some bacteria possess multiple T6SS gene clusters, each of which may have distinct functions and be subject to different regulations (28, 30, 31).

*Acidovorax citrulli* is the causal agent of bacterial fruit blotch (BFB), a seed-borne bacterial disease of cucurbit crops that causes significant economic losses worldwide (32). Several molecular apparatuses, including type Ⅱ secretion system (T2SS), type Ⅲ secretion system (T3SS), type Ⅳ pili (T4P), and polar flagellum, have been found to contribute to the pathogenicity of *A. citrulli* (33 - 37). The T3SS is known to be essential to pathogenicity as the T3SS defective mutants fail to cause disease in host plants and hypersensitive response in nonhost plants (34, 38). However, the extent to which the T3SS influences the interplay between *A. citrulli* and plant-associated microbes, and the potential impact of such interactions on virulence, remains largely unexplored. In addition, it has been shown that there are at least two genetically and physiologically distinct groups in *A. citrulli*; group I strains are mainly isolated from nonwatermelon hosts, and group Ⅱ strains are mostly from watermelon hosts (32, 39). Notably, T3SS effectors exhibit a differential distribution in these two groups, suggesting that *A. citrulli* from distinct lineages may possess unique preferences for host plants (40). The T6SS has also been implicated in host-plant interaction since T6SS mutants of a group I strain xjl12 show significantly decreased biofilm formation and pathogen transmission from seed to seedling in melon (41). Our recent study has demonstrated that a group Ⅱ *A. citrulli* strain AAC00-1 has an active T6SS with potent killing abilities against both eukaryotic and prokaryotic competitors (24). However, the T6SS function in pathogenicity and microbial competition in plants remain to be established.

In this study, we aim to elucidate the roles of *A. citrulli* T3SS and T6SS in the interaction between plants and microbes. Using RNA-seq transcriptome analyses, we identified key gene pathways that are modulated in watermelon plants in response to bacterial T3SS and T6SS during infection. We demonstrated that a constructed *A. citrulli* (Ac_av_) mutant, with inactive T3SS but a functional T6SS, is nonpathogenic to plants and capable of killing several pathogenic bacteria through T6SS-mediated mechanisms. These findings suggest an effective strategy for controlling plant-associated diseases and promoting food safety and human health.

## RESULTS

### T3SS and T6SS play different roles in the virulence and proliferation of *A. citrulli*

**Fig 1:**
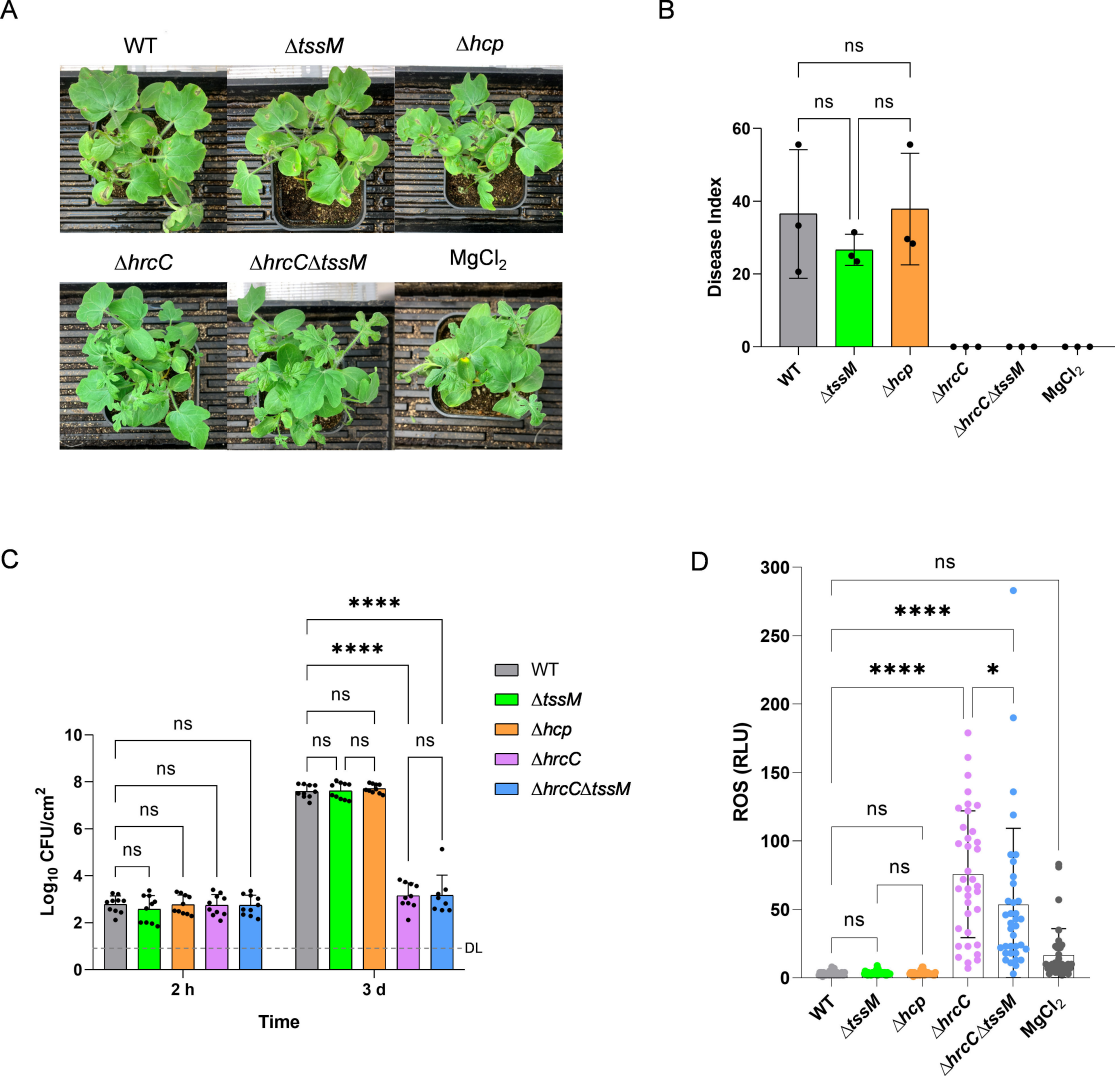
Effect of T6SS and T3SS on the virulence and bacterial proliferation of *A. citrulli* in watermelon. (**A**) The appearance of watermelon seedlings at 7 dpi. MgCl_2_ (10 mM) was sprayed as the negative control. (**B**) The disease index was calculated at 7 dpi as described in Materials and Methods. The error bars represent the standard deviation of the means from three experiments, each containing six inoculated watermelon seedlings per tested strain. (**C**) The proliferation of *A. citrulli* strains on watermelon seedlings. The error bars represent the standard deviation of the means from two independent experiments, each containing five replicates. (**D**) Effect of T6SS and T3SS on inducing ROS burst on *N. benthamiana*. The bacteria at 10^8^ CFU/mL were infiltrated into *N. benthamiana* leaves, and ROS production was assessed by using L-102 chemiluminescence at 15 hpi. The error bars represent the standard deviation of the means from three independent experiments, each containing 12 replicates per treatment. WT, *A. citrulli* AAC00-1 wild type; ∆*tssM* and ∆*hcp*, T6SS-null strains; ∆*hrcC*, T3SS-null strain; ∆*hrcC*∆*tssM*, a mutant that both T3SS and T6SS are inactive. Statistical significance was calculated by one-way (**B, D**) or two-way (**C**) ANOVA with Tukey’s multiple comparisons test. * *P* < 0.05, **** *P* < 0.0001; ns, not significant; DL, detection limit.

To investigate whether T6SS contributes to the virulence of *A. citrulli*, we constructed a series of T6SS and T3SS mutants, including two T6SS mutants ∆*hcp* and ∆*tssM*, lacking the tube protein and the inner membrane protein, respectively, and a T3SS mutant ∆*hrcC*, lacking the gene encoding the T3SS outer-membrane-ring protein (20, 42, 43). We then applied a seedling inoculation assay to test the virulence of WT and these mutants. The disease symptoms of watermelon in different treatments were compared after 7 days postinoculation (dpi). Neither the T3SS-null strain ∆*hrcC* nor the T3SS/T6SS double-deletion strain (∆*hrcC*∆*tssM*) could cause BFB symptoms in plants (Fig. 1A). In contrast, the T6SS-null strains, ∆*tssM* and ∆*hcp*, were still able to induce BFB in plants, with no significant difference from the WT strain (Fig. 1B). Therefore, the T3SS is the main virulence factor of *A. citrulli*, which is consistent with previous finding (38, 44, 45).

To examine the contribution of T3SS and T6SS to the proliferation of *A. citrulli* in watermelon seedlings and determine whether T6SS inactive strains are impaired in growth *in planta*, we infiltrated cell suspensions containing *A. citrulli* WT and mutant strains at a concentration of approximately 10^5^ CFU/mL into the watermelon cotyledons (Fig. S1). The number of WT cells increased significantly from 6.06 × 10^2^ CFU/mL (2 h postinoculation, hpi) to 3.96 × 10^7^ CFU/mL (3 dpi), which was also observed in the two T6SS-null strains, ∆*tssM* and ∆*hcp* (Fig. 1C). In contrast, plants infiltrated with ∆*hrcC* or ∆*hrcC*∆*tssM* did not show any BFB symptoms at 3 dpi, and the cell count of these two mutants only had a minor increase between 2 hpi and 3 dpi (from 5.63 × 10^2^ to 1.42 × 10^3^ CFU/mL for ∆*hrcC* and 5.54 × 10^2^ to 1.46 × 10^3^ CFU/mL for ∆*hrcC*∆*tssM*, respectively) (Fig. 1C). These results suggest that while the proliferation of the T3SS-null mutant and the double-deletion mutant was restricted by the plant host, the T6SS-null mutants were not affected.

The production of reactive oxygen species (ROS) is a hallmark of phytopathogen infection and initiation of plant defense (46, 47). We, therefore, tested the ROS production of *Nicotiana benthamiana* plants infiltrated with different T3SS/T6SS mutants of *A. citrulli*. Plants infiltrated with ∆*hrcC* or ∆*hrcC*∆*tssM* showed a significant ROS burst (Fig. 1D). In contrast, plants infiltrated with ∆*tssM* or ∆*hcp* showed almost no ROS production, similar to those plants infiltrated with WT (Fig. 1D). These results suggest that the T3SS is crucial for the interaction between *A. citrulli* and the host plant, while the T6SS does not play a significant role in ROS production during the infection.

### Inactivation of T6SS reduces the competition ability of *A. citrulli*

**Fig 2:**
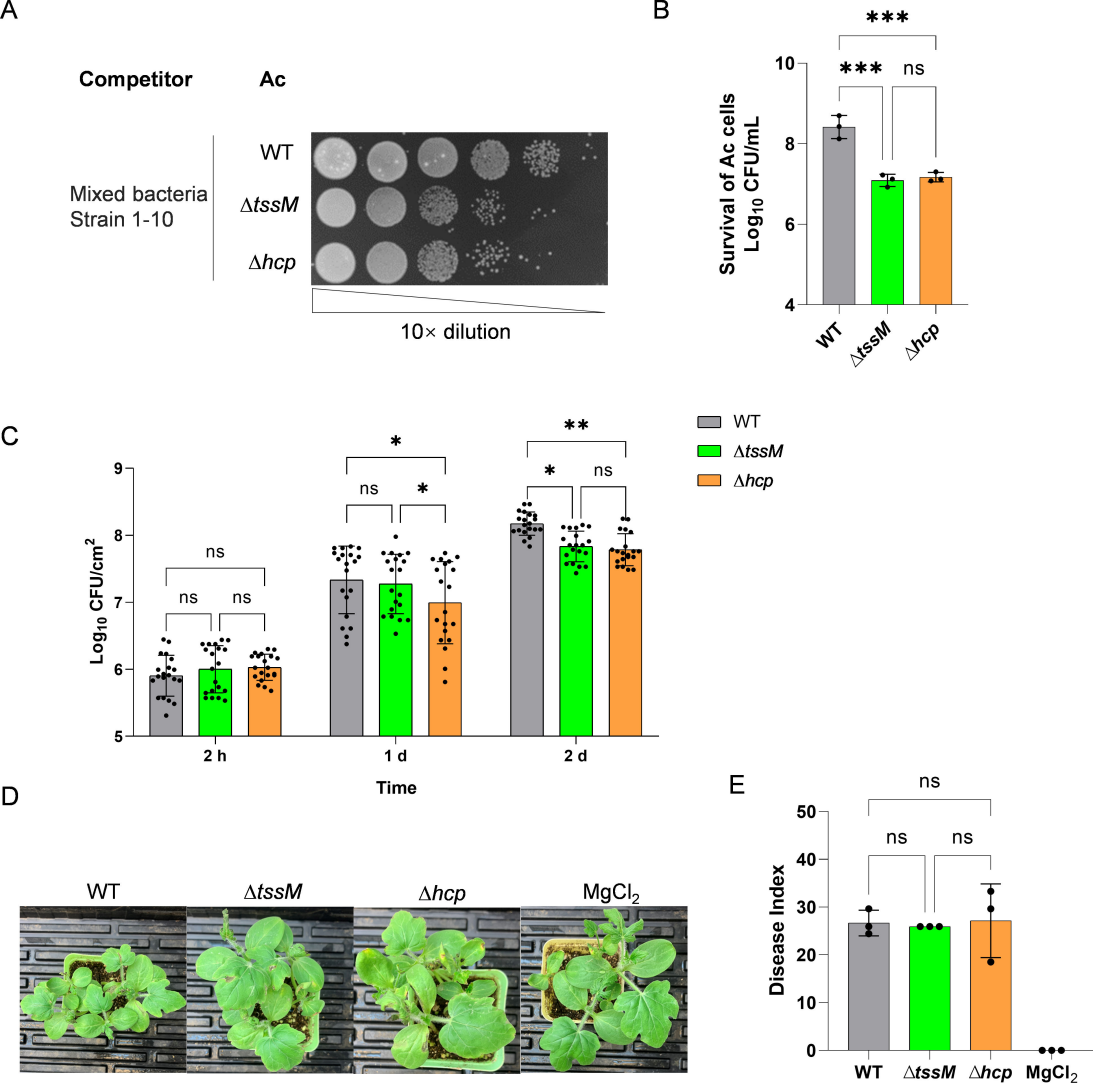
Effect of T6SS on competition ability and virulence of *A. citrulli* (Ac) mixed with the phyllosphere bacteria. (**A**) The appearance of survival of *A. citrulli* strains after being coincubated with the phyllosphere bacteria for 24 h. (**B**) Survival of *A. citrulli* cells after being coincubated with the phyllosphere bacteria for 24 h *in vitro* (Ac:Competitor_mix_ = OD10:10). The error bars represent the standard deviation of the means from three independent experiments. (**C**) Survival of *A. citrulli* cells after being coinfiltrated with the phyllosphere bacteria on watermelon cotyledons at different time (Ac:Competitor_mix_ = OD1:10). The error bars represent the standard deviation of the means from two independent experiments, each containing five replicates. (**D**) The appearance of watermelon seedlings 7 dpi of the mixed bacterial suspension of *A. citrulli* strains and phyllosphere bacteria (Ac:Competitor_mix_ = OD1:1), MgCl_2_ (10 mM) was sprayed as the negative control. (**E**) Disease of coinfection with *A. citrulli* strains and phyllosphere bacteria at 7 dpi. The disease index was calculated as described in Materials and Methods. The error bars represent the standard deviation of the means from three experiments, each containing six inoculated watermelon seedlings per tested strain. WT, *A. citrulli* AAC00-1 wild type; ∆*tssM* and ∆*hcp*, T6SS-null strains. Statistical significance was calculated by one-way (**B, E**) or two-way (**C**) ANOVA with Tukey’s multiple comparisons test. * *P* < 0.05, ** *P* < 0.01, *** *P* < 0.001; ns, not significant.

Next, we tested the role of the T6SS of *A. citrulli* in competition with other bacterial species by isolating the phyllosphere bacteria of watermelon. We identified 10 bacterial strains with different colony phenotypes from seven different species (Table 1; Fig. S2). To mimic the natural competition, we used a mixture of these 10 strains as competitors to compete with WT and the T6SS-null strains both *in vitro* and *in vivo*. Results showed that the survival of both ∆*tssM* and ∆*hcp* significantly decreased compared to the wild type when coincubated with the mixed bacteria for 24 h on the LB agar plate (Fig. 2A and B), whereas no difference was observed when coincubated for 6 h (Fig. S3A and B). No significant difference was observed between the T6SS-null strains, ∆*tssM* and ∆*hcp,* with either 6 h or 24 h treatments (Fig. S3A and B; Fig. 2A and B). These results suggest that the T6SS-inactive strains of *A. citrulli* are impaired in interspecies competition. A similar result was obtained *in vivo* with watermelon seedlings. With a concentration at approximately 10^8^ CFU/mL of *A. citrulli* in the mixed suspension (Fig. S3C), bacteria were isolated from the infiltrated watermelon cotyledons at 2 hpi, 1 dpi, and 2 dpi. Both ∆*tssM* and ∆*hcp* significantly reduced at 2 dpi compared to the WT (Fig. 2C). These results indicate that the inactivation of T6SS decreases the survival ability of *A. citrulli* both *in vitro* and *in vivo* when competing against the watermelon phyllosphere bacteria.

**TABLE 1:** Phyllosphere bacteria isolated from watermelon seedlings^*a*^

No.	Isolate	Blast results according to 16S rDNA sequence
1		*Enterobacter ludwigii*
2	1)	*Enterobacter cloacae*
	2)	*E. cloacae*
3		*Pseudomonas* sp. 13159349
4	1)	*Stenotrophomonas maltophilia*
	2)	*S. maltophilia*
5		*Bacillus cereus*
6		*Microbacterium* sp. strain
7	1)	*Pseudomonas otitidis*
	2)	*P. otitidis*

^*a*^ No., sample number of the isolations.

Notably, no symptoms of BFB were observed in watermelon seedlings treated with a concentrated mixture of phyllosphere bacteria (10^9^ CFU/mL) and either *A. citrulli* WT or mutant strains at 7 dpi (Fig. 2D and E). These findings indicate that the phyllosphere bacteria may provide protection to the seedlings against *A. citrulli* infections and suggest an evolutionary pressure for selecting T6SS-mediated competition in *A. citrulli*.

### Distinct plant cellular responses to T6SS and T3SS validate that the T3SS dictates virulence

To determine the role of the T6SS in the interaction of *A. citrulli* and plant, we analyzed the transcriptome of watermelon infiltrated with *A. citrulli* WT strain, the T3SS-null strain ∆*hrcC*, the T6SS-null strain ∆*tssM*, and the double-deletion mutant ∆*hrcC*∆*tssM*. The general transcriptome analysis of each sample in different treatments was shown in Fig. S4A and B. With a filter at a |Log_2_ fold change| cutoff of 1 and a *P*-value of 0.05, we identified a total of 3,038; 803; and 4,549 differentially expressed genes (DEGs) in the ∆*hrcC*, ∆*tssM*, and ∆*hrcC*∆*tssM* treated samples compared to the WT infiltrated sample, respectively (Fig. S4C and D). The comparison group of ∆*hrcC*_vs_WT and ∆*hrcC*∆*tssM*_vs_∆*tssM*, as well as the group of ∆*tssM*_vs_WT and ∆*hrcC*∆*tssM*_vs_∆*hrcC*, showed a similar number of DEGs (Fig. S4C and D). The RNA-seq results were validated by quantitative real-time PCR (qRT-PCR) with 19 genes involved in different pathways (Fig. S5).

**Fig 3:**
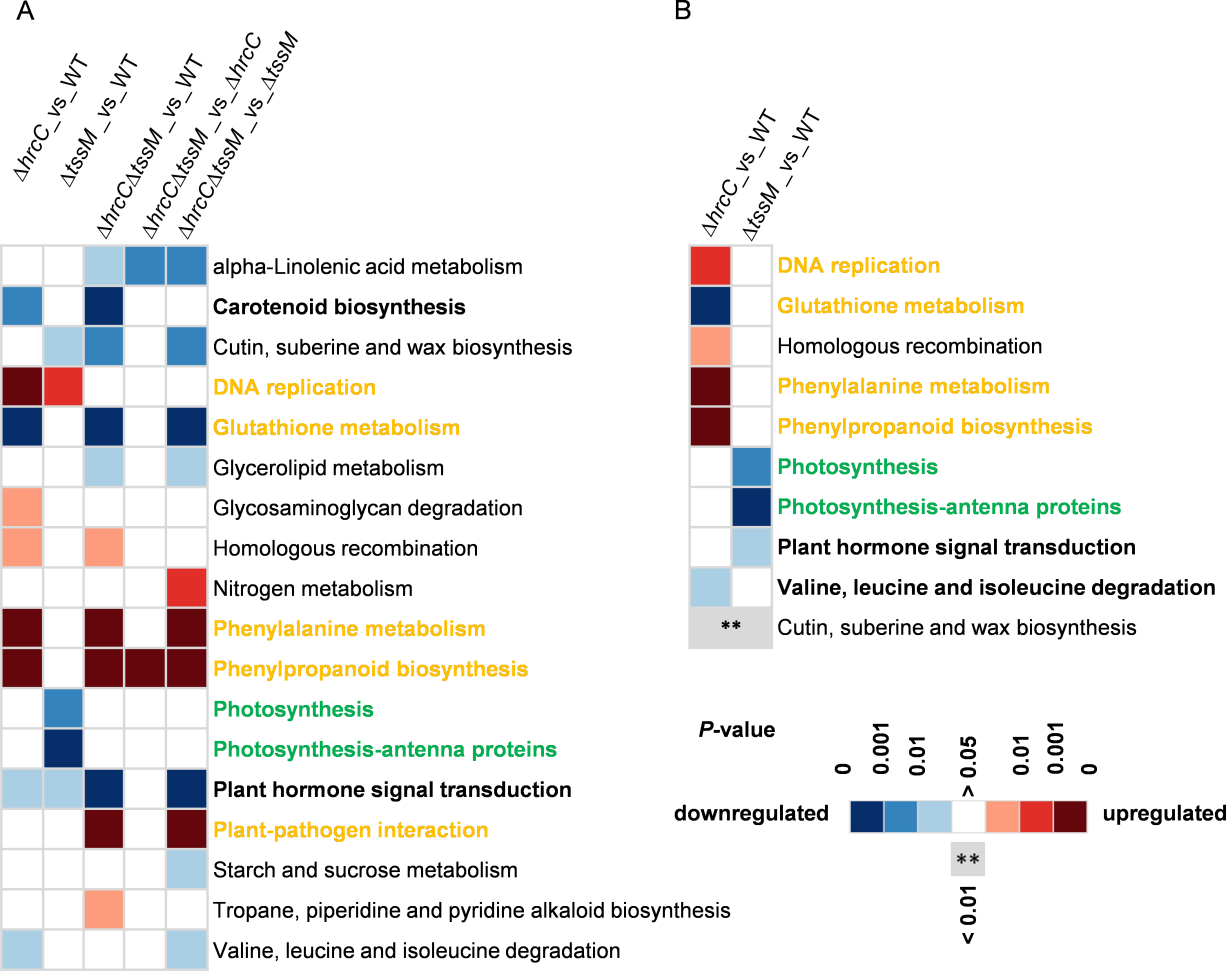
Heat map of *P*-value (adjusted) for KEGG (Kyoto Encyclopedia of Genes and Genomes) pathways representing differentially expressed genes in different comparisons. (**A**) KEGG pathways enriched from the differentially expressed genes affected by the T3SS or T6SS. (**B**) KEGG pathways enriched from the differentially expressed genes affected by T3SS or T6SS alone. Blue and red cells represent significantly downregulated and upregulated, respectively (*P* < 0.05). White cells represent no significantly differentially expressed (*P* > 0.05). Grey cell with * inside represents this KEGG pathway was significantly enriched from the shared differentially expressed genes affected by both T3SS and T6SS. ** *P* < 0.01. WT, *A. citrulli* AAC00-1 wild type; ∆*tssM*, T6SS-null strain; ∆*hrcC*, T3SS-null strain; ∆*hrcC*∆*tssM*, a mutant that both T3SS and T6SS are inactive. Pathways in bold and colored orange or green represent the most significant pathways triggered by T3SS or T6SS of *A. citrulli*, respectively. Pathways in bold and black represent other significantly enriched pathways during infection.

**Fig 4:**
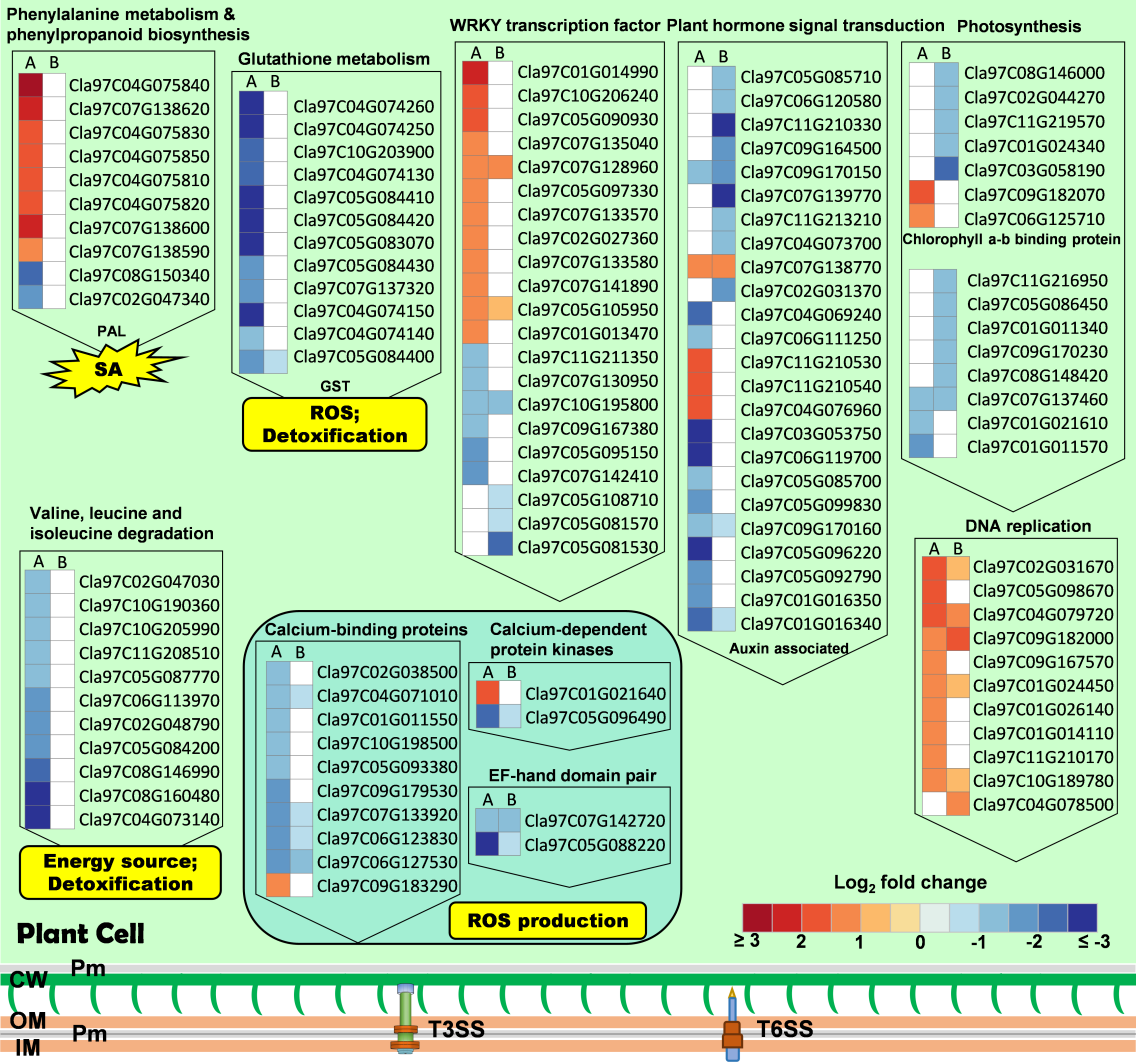
Heat map of Log_2_ fold change of differentially expressed genes (DEGs) triggered by T3SS (**A**) and T6SS (**B**) of *A. citrulli* in the key KEGG (Kyoto Encyclopedia of Genes and Genomes) pathways according to the RNA-Seq data. DEGs were filtered at a cutoff of |Log_2_ fold change| > 1 and *P*-value <0.05 by DESeq2 R package (1.20.0). The comparison groups represent plants infiltrated with: A, ∆*hrcC*_vs_WT; B, ∆*tssM*_vs_WT. WT, *A. citrulli* AAC00-1 wild type; ∆*tssM*, T6SS-null strain; ∆*hrcC*, T3SS-null strain. Pm, periplasm; CW, cell wall; OM, outer membrane; IM, inner membrane. White cells represent not significantly differentially expressed. Red and blue cells represent the genes that were significantly upregulated or downregulated, respectively.

There were 803 genes differentially expressed in the T6SS-null strain ∆*tssM* infiltrated samples compared to the WT infiltrated samples (Table S1). These DEGs were significantly enriched in the pathways of photosynthesis-antenna proteins, photosynthesis, cutin, suberine and wax biosynthesis, and plant hormone signal transduction (Fig. 3A; Fig. S6A). In particular, five genes encoding chlorophyll *a*/*b* binding proteins were identified with a fold change ranging from 0.19 to 0.48 in the ∆*tssM* infiltrated samples compared to the WT infiltrated samples (Fig. 4; Table S1). Interestingly, photosynthesis-associated genes were not differentially expressed in ∆*hrcC*-inoculated plants, except Cla97C07G137460, which had a similar downregulated expression level with that in the ∆*tssM* treated samples (Fig. 4). According to the GO enrichment analysis, the DEGs influenced by T6SS were mainly classified in the photosynthesis-associated terms of the “cell component” category, including photosystem, photosynthetic membrane, and thylakoid (Fig. S6B).

There were 2,615 DEGs triggered solely by T3SS and 380 by T6SS, and 423 DEGs were triggered by both T3SS and T6SS (Fig. S4D). The 380 T6SS-mediated DEGs (146 genes upregulated and 234 downregulated) were found to be enriched in the pathways of photosynthesis and plant hormone signal transduction (Fig. 3B). In contrast, the 1,057 upregulated genes influenced only by T3SS were enriched in pathways associated with phenylalanine metabolism, phenylpropanoid biosynthesis, DNA replication, and homologous recombination, while the 1,558 downregulated genes were enriched in glutathione metabolism and valine, leucine, and isoleucine degradation pathways (Fig. 3B). Lastly, the 423 shared genes affected by both T3SS and T6SS were significantly enriched in the cutin, suberine, and wax biosynthesis pathway (Fig. 3B).

### T3SS has little effect on T6SS-mediated competition against pathogenic bacteria

**Fig 5:**
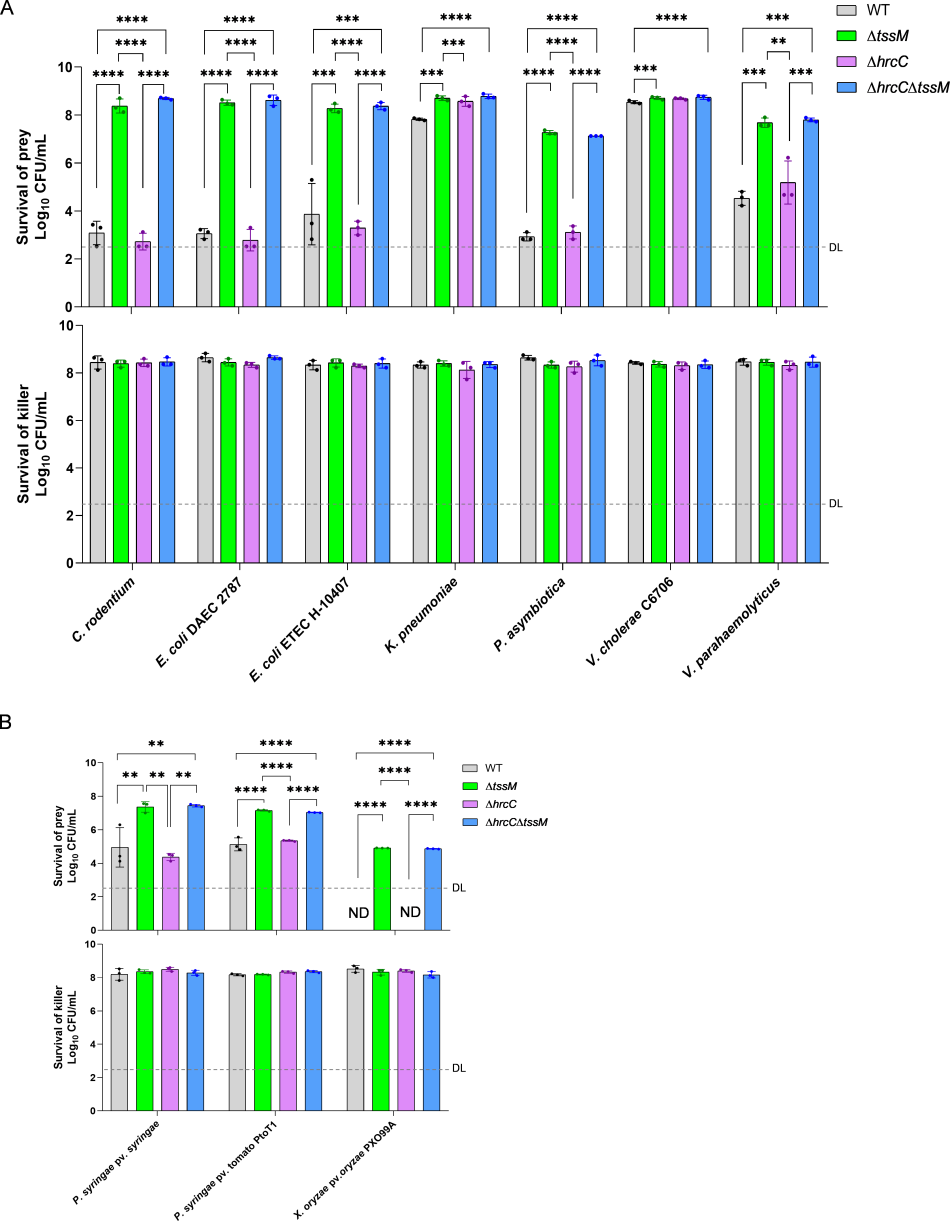
T6SS-dependent competition against bacterial pathogens. (**A**) Competition with pathogens that may contaminate edible plants. (**B**) Competition with important plant pathogens. For both (**A**) and (**B**), the error bars represent the standard deviation of the means from three experiments. WT, *A. citrulli* AAC00-1 wild type; ∆*tssM*, T6SS-null strain; ∆*hrcC*, T3SS-null strain; ∆*hrcC*∆*tssM*, a mutant that both T3SS and T6SS are inactive. Cells of different strains were selected by different antibiotics (see Table S2, *A. citrulli* cells were selected with gentamicin, 20 µg/mL). Statistical significance was calculated by one-way ANOVA with Tukey’s multiple comparisons test. ** *P* < 0.01, *** *P* < 0.001, **** *P* < 0.0001; ND, not detectable; DL, detection limit.

We have previously demonstrated that *A. citrulli* exhibits potent T6SS-mediated bacterial killing abilities against a diverse range of bacterial pathogens (24). However, whether the T3SS has any effect on the T6SS functions is unclear. Therefore, we tested the survival of seven human pathogens and three plant pathogens as prey in a competition assay against *A. citrulli* WT and T3SS/T6SS mutants. The results showed that *A. citrulli* with active T6SS had a strong killing ability for all the pathogenic bacteria except for *Klebsiella pneumoniae* and *Vibrio cholerae* (C6706) (Fig. 5A and B). Furthermore, the T3SS-null mutant ∆*hrcC* displayed equivalent bactericidal activity to WT, indicating that T6SS-based killing is independent of T3SS activity.

### T3SS-null avirulent *A. citrulli* can protect plants from a plant pathogen

**Fig 6:**
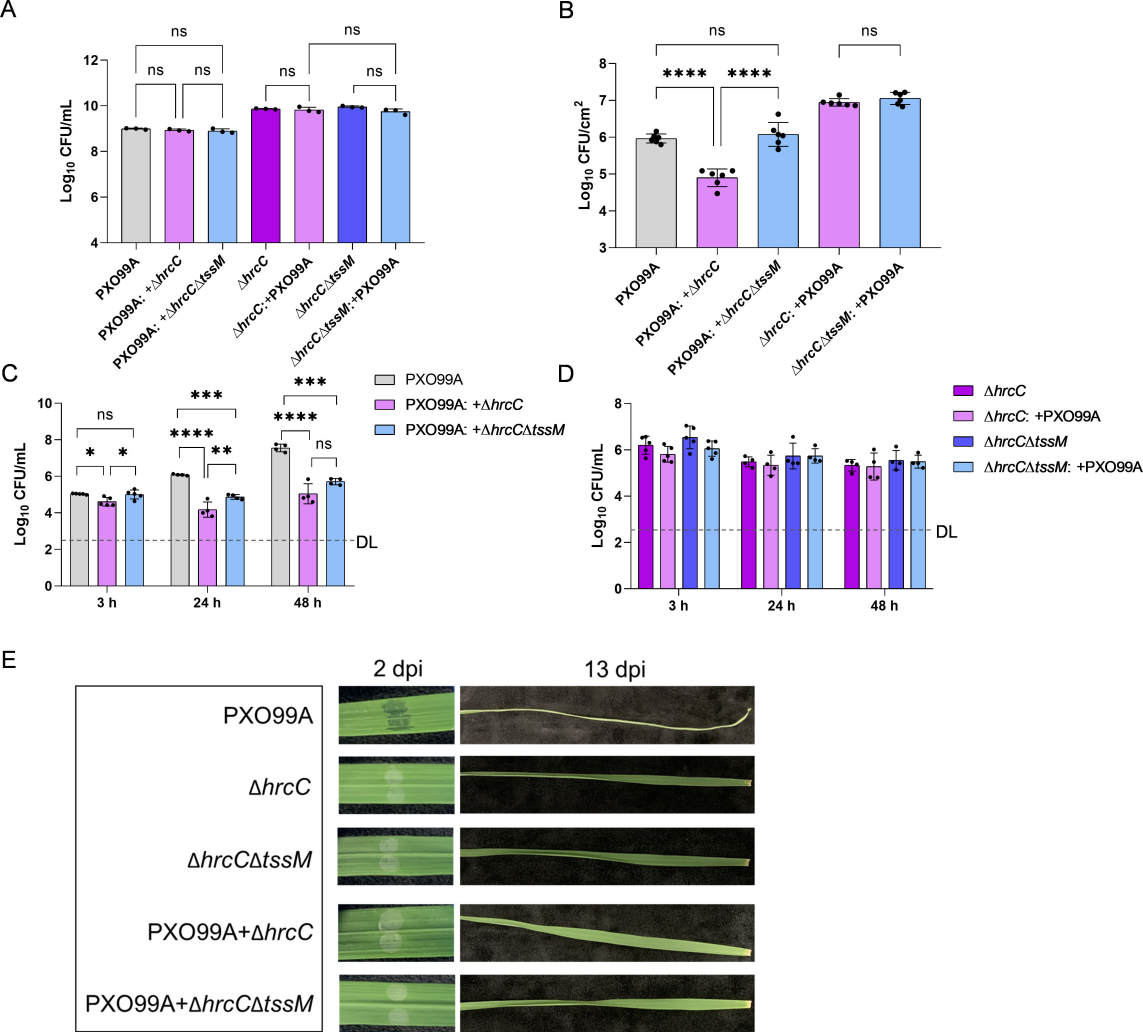
T6SS-based competition results against *X. oryzae* pv. *oryzae* PXO99A in *N. benthamiana* and rice. (**A**) Concentrations of initial bacterial suspension for infection. (**B**) Bacteria isolated from the inoculation site of *N. benthamiana* and selected by antibiotics at 3 hpi. The last two columns show no significant difference between the cell number of ∆*hrcC* and ∆*hrcC*∆*tssM*. (**C**) PXO99A was isolated from the inoculation site of rice and selected by kanamycin after being infiltrated at different time points. (**D**) *A. citrulli was* isolated from the inoculation site of rice and selected by gentamicin after being infiltrated at different time points. (**E**) Disease symptoms of rice at 2 dpi by infiltration and 13 dpi by scissor cutting. WT, *A. citrulli* AAC00-1 wild type; ∆*hrcC*, T3SS-null strain; ∆*hrcC*∆*tssM*, a mutant that both T3SS and T6SS are inactive. Statistical significance was calculated by one-way ANOVA with Tukey’s multiple comparisons test. * *P* < 0.05, ** *P* < 0.01, *** *P* < 0.001, **** *P* < 0.0001; ns, not significant; DL, detection limit.

Now that we have demonstrated the *A. citrulli* T3SS-null mutant ∆*hrcC* can efficiently eliminate *X. oryzae* pv. *oryzae* PXO99A, a highly important plant pathogen (Fig. 5B), we next examined the role of T6SS in inhibiting *X. oryzae* pv. *oryzae* infection in *N. benthamiana* and rice plants. The T3SS/T6SS double-deletion strain ∆*hrcC*∆*tssM* was used as a control. The initial concentration of PXO99A or PXO99A mixed with different *A. citrulli* strains for infection was shown in Fig. 6A. After infiltration for 3 h in *N. benthamiana*, the ∆*hrcC* mutant significantly inhibited the growth of PXO99A, while the ∆*hrcC*∆*tssM* strain had no effect on PXO99A cells (Fig. 6B). In rice plant, the ∆*hrcC* showed T6SS-dependent inhibition at 24 h but not at 48 h (Fig. 6C). In addition, survival of PXO99A cells in both treatments was significantly less than that in the control which was only infiltrated with PXO99A (Fig. 6C), and survival of *A. citrulli* appeared to be stably maintained (Fig. 6D). These results indicate that the presence of *A. citrulli* could inhibit PXO99A growth, and the T6SS mainly functions during the early stage of infection.

We also investigated the disease development of bacterial blight of rice infected with PXO99A in combination with either T6SS-active (∆*hrcC*) or T6SS-null (∆*hrcC*∆*tssM*) *A. citrulli* cells. The leaves coinfected with PXO99A mixed with ∆*hrcC* or ∆*hrcC*∆*tssM* showed no disease symptoms by infiltration (2 dpi) and cutting the leaves (13 dpi) (Fig. 6E). In contrast, the PXO99A-only infection control showed obvious water spots by infiltration and the infected leaves were dehydrated upon cutting at 13 dpi. Taken together, our findings suggest that the *A. citrulli* ∆*hrcC* is avirulent and capable of protecting rice from bacterial blight, albeit the protection may be independent of T6SS activity under the tested conditions.

## DISCUSSION

Phytobacteria live in a complex environment, facing various stresses from competing microbes in the same niche and from the innate immunity of plants. As a result, bacteria use specialized secretion systems to modulate their environment within a host or around other microbes (48 - 50). In this study, we delineated the contributions of T3SS and T6SS to virulence and competitive fitness of the BFB-causing pathogen *A. citrulli*. Using transcriptome analysis, genetic mutations, and *in vitro* and *in planta* competitions, we report that the T6SS mediates interbacterial competition and the T3SS dictates host infection, and the inactivation of T3SS has little effect on T6SS functions. We further show that the T3SS-inactivated *A. citrulli* strain could outcompete a number of plant-associated bacteria and protect rice from *X. oryzae* infection. These data demonstrate an effective strategy to transform *A. citrulli* from plant infection to plant protection.

The T6SS has been implicated in BFB seed-to-seedling transmission in an *A. citrulli* group I strain xjl12, in which several deletion mutants of T6SS genes exhibit significantly decreased biofilm formation (41). In contrast, we performed similar biofilm assays in *A. citrulli* group II strain AAC00-1 and found that deletion in T6SS genes promoted biofilm formation (Fig. S8), suggesting that strain backgrounds may play a role in phenotypic variations (40). Nonetheless, both studies demonstrate that the T6SS-null mutants are capable of inducing WT-comparable BFB symptoms, further supporting that the T6SS does not contribute to virulence in host plant.

Our analysis of the transcriptome provides insights into how plant cells respond to the activities of T3SS and T6SS at the cellular level. The T3SS suppresses the expression of genes involved in phenylalanine metabolism, phenylpropanoid biosynthesis, DNA replication, and homologous recombination in the plant. Phenylalanine is an intermediate aromatic amino acid in the salicylic acid biosynthesis pathway, and phenylpropanoids, which are produced from phenylalanine, are a large class of secondary metabolites (51). These phenylpropanoids are involved in responses to various biotic and abiotic stimuli and are also crucial for the defense system in the plant (51, 52). T3SS induces gene expression in glutathione metabolism; carotenoid biosynthesis; valine, leucine, and isoleucine degradation; and plant hormone signal transduction. Glutathione and glutathione *S*-transferases (GST) are important in the immune system of plants as they are involved in the defense against bacterial, fungal, and viral pathogens (53, 54). In addition, GST also has the function of detoxification by conjugation with glutathione as well as hormone transport (55). Genes in the plant-pathogen interaction and MAPK signaling pathway plant, as well as plant hormone signal transduction, cutin, suberine and wax biosynthesis pathways, were affected by both T3SS and T6SS. Genes in the photosynthesis pathway were specifically affected by the T6SS. Notably, when the T3SS and the T6SS were both inactivated, more DEGs were identified than their individual mutants, suggesting a complex additive effect between T3SS- and T6SS-mediated cellular responses during *A. citrulli* infection.

As a lethal molecular weapon against neighboring cells, the T6SS has been found to have a number of different functions, including interspecies competition, nutrient acquisition, and interaction with different hosts (17, 18, 56, 57). However, its application as a biocontrol agent is limited because bacterial competition assays can be affected by nutrients, temperature, cations, and other environmental conditions (23, 58). In a recent study, Bernal et al. found that *P. putida* could eradicate *X. campestris in vivo* and reduce necrosis in *N. benthamiana* (59), suggesting the potential for novel biocontrol agents with strong T6SS-based killing abilities. In this study, we constructed an *A. citrulli* avirulent Ac_av_ strain with a T3SS-null mutation, which exhibits potent T6SS-dependent bactericidal activities against a number of pathogenic bacteria that may infect the plants or transmit to humans through contamination of edible plants. Results of coinfection experiments with *X. oryzae* pv. *oryzae* and the *A. citrulli* Ac_av_ show that the disease symptoms of bacterial blight on rice were eliminated by the *A. citrulli* avirulent strains. These data collectively suggest that *A. citrulli* Ac_av_ is an effective and promising tool for managing plant infections and promoting plant and human health in the future.

## MATERIALS AND METHODS

### Bacterial strains and plasmids

The strains and plasmids used in this study are described in Table S2. Bacteria were grown in LB (w/v, 1% tryptone, 0.5% yeast extract, 0.5% NaCl) at 28°C aerobically. Antibiotics were used in the following concentrations: kanamycin (50 µg/mL), gentamicin (20 µg/mL), and streptomycin (100 µg/mL).

### Construction of mutant strains

Unmarked deletion mutants were generated using the *sacB*-based homologous recombination method described previously (45). Approximately 1 kb of each upstream and downstream region of the desired gene was amplified from the genome DNA of wild-type strain AAC00-1. The PCR fragments were ligated into pEXG2.0 or pK18*mobsacB* to create the suicide plasmid of each desired gene (Table S2). After confirmation by sequencing, the plasmid was introduced into the *A. citrulli* strain AAC00-1 by conjugation. The mutant strains were confirmed by PCR. All the primers are listed in Table S2.

### Biofilm formation assays

The biofilm formation was determined as Tian et al. described using 12-well plates (41). Overnight growing suspension of WT and mutants were adjusted to OD_600_ = 1.0. A diluted suspension was transformed into a 12-well plate at the ratio of 1:100 with LB broth and incubated at 28°C for 48 h without agitation. The LB broth was used as the negative control. Cultures were discarded and the plates were submerged in distilled H_2_O three times. The plate was dried at 37°C before being stained with 1% crystal violet for 45 min. Then, the crystal violet was removed, and the plate was submerged in distilled H_2_O three times. The biofilm was dissolved in 95% ethanol for 2 h, and the absorbances were measured at 590 nm using a Synergy H1 Hybrid Microplate Reader (BioTek Instruments, USA). *P*-values were calculated by one-way ANOVA with Tukey’s multiple comparisons test.

### Bacterial isolation from plant

For phyllosphere microbe isolation, 21-day-old watermelon seedlings (*Citrullus lanatus* Jingxin No. 2, provided by the Beijing Academy of Agriculture and Forestry Sciences, Beijing, China) grown in the greenhouse were used. Both cotyledons and true leaves were frozen in liquid nitrogen and ground into powder at 45 Hz for 100 s with the Tissue Lyser Wonbio-L (Shanghai Wonbio Biotechnology, China). One milliliter of 0.85% NaCl was added and mixed well. The homogenate was streaked on LB plates and incubated at 28°C for 48 h. Colonies with different phenotypes were identified according to the sequence of their 16S rDNA.

For the proliferation evaluation, the overnight-grown *A. citrulli* WT strains and different mutants were adjusted to OD_600_ = 0.2 (~10^8^ CFU/mL) with 10 mM MgCl_2_. The suspension was diluted 1,000 times and infiltrated with the cotyledons of 14-day-old watermelon (Jingxin No. 2) seedlings. Twenty-five leaf dishes (5 mm in diameter) from five infiltrated leaves of different plants were collected after being infiltrated at different time. The leaf dishes were separated as five samples and ground as above. One milliliter of 0.85% NaCl was added, and vortex mixed to make a homogenate. Cells of each strain were enumerated by a series of 10-fold dilutions and plating on agar plates after being incubated at 28°C for 48 h. There were two independent experiments with five replicates in each of the treatments. A two-way ANOVA with Tukey’s multiple comparisons test was used to determine the *P*-values between the different treatments.

### Bacterial competition assays

For *in vitro* competition assay of phyllosphere bacteria, different strains were grown overnight in LB broth with appropriate antibiotics. Cells were centrifuged at 10,000 *g* for 3 min and resuspended in fresh LB to make a suspension of OD_600_= 10. The 10 strains isolated from watermelon seedlings were mixed together at a ratio of 1:1 as the competitor. Competitor and different strains of *A. citrulli* cells (wild-type strain and two T6SS deletion mutants, ∆*tssM* and ∆*hcp*) were mixed at a ratio of 1:1, and an aliquot of 10 µL suspension was spotted on LB plates and incubated at 28°C. After incubation for 6 h and 24 h, cells were resuspended in 500 µL LB. A series of 10-fold dilutions were plated on LB plates with kanamycin and enumerated after being incubated at 28°C for 48 h. For the competition assay against pathogenic bacteria, competitor (*A. citrulli* cells, Gm^R^) and prey cells (different pathogenic bacterial cells, resistant to different antibiotics, see Table S2) were mixed at 1:1 (competitor OD_600_ = 10, prey OD_600_ = 1). After incubation for 3 h at 28°C (*A. citrulli* strains compete against *Photorhabdus asymbiotica*, *V*. *parahaemolyticus*, *P. syringae* pv. *syringae*, and *X. oryzae* pv. *oryzae* PXO99A) or 37°C (*A. citrulli* strains compete against other bacteria in this study), the bacterial cells were selected by antibiotics and enumerated as described. All the experiments were repeated three times. A mean value was calculated, and a one-way ANOVA with Tukey’s multiple comparisons test was used to determine the *P*-values between the different treatments.

For the *in vivo* competition assay of pathogenic bacteria, bacteria of different strains were grown overnight in LB broth with (Ac cells) or without (phyllosphere bacteria) appropriate antibiotics. Cells were centrifuged at 10,000 *g* for 3 min and resuspended in 10 mM MgCl_2_ to make a suspension of OD_600_ = 10. The 10 strains isolated from the watermelon seedlings were mixed together at a ratio of 1:1 as the competitor. For the three *A. citrulli* strains, a final concentration of OD_600_ = 1 was used, just in case of severe disease on the infiltrated leaves. Competitor and different *A. citrulli* cells were mixed at the ratio of 1:1 and infiltrated into the cotyledons of 14-day-old watermelon (Jingxin No. 2) seedlings. Bacterial cells were isolated and enumerated at different time points after the infiltration. The method for the *A. citrulli* isolation was the same as the proliferation evaluation assay described above. For the *in vivo* competition assay against *X. oryzae* pv. *oryzae* PXO99A, competitor (*A. citrulli*, Gm^R^) and prey (PXO99A, Km^R^) cells were mixed at a ratio of 1:1 (competitor OD_600_ = 10, prey OD_600_ = 1) and infiltrated with 21-day-old rice plant; bacterial cells were isolated at different time points and enumerated on LB plates with different antibiotics. There were two independent experiments with at least four replicates in each of them. A two-way ANOVA with Tukey’s multiple comparisons test was used to determine the *P*-values between the different treatments. Meanwhile, rice leaves were inoculated with the scissor clipping method (60) to see whether the bacterial competition *in vivo* would influence the development of the disease.

### Virulence assays

The virulence of different *A. citrulli* strains or the mixed bacteria was tested on 21-day-old watermelon seedlings (Jingxin No. 2). Pathogenicity tests of WT and different mutants were investigated as previously described with a minor modification (61). Briefly, the overnight-grown (12 h) *A. citrulli* cells in LB broth were adjusted to OD_600_ = 0.2 (~10^8^ CFU/mL) with 10 mM MgCl_2_. Then, watermelon seedlings were sprayed until runoff with different cell suspensions. Seedlings per treatment were kept in a plastic bag to prevent cross-contamination and keep the humidity. Disease symptoms were evaluated at 7 dpi using a 0–9 disease severity scale: 0, no symptoms; 1, 3, 5, and 7, necrotic lesions on approximately 25%, 50%, 75%, and 100% of the leaves, respectively; and 9 represents total death of the seedling. The disease index (DI) was calculated based on the formula: DI = ∑(A × B) × 100 ∕ ∑C × 9, where A is the severity scale (0, 1, 3, 5, 7, or 9), B is the number of plants showing that scale per treatment, and C is the total number of seedlings in that treatment.

For the virulence test of the mixed bacterial cells, the final concentration of WT and mutants was OD_600_ = 1. The 10 strains isolated from watermelon seedlings were also adjusted to OD_600_ = 1. The inoculation method and the disease severity evaluation were the same as described above. All the experiments were repeated three times, and a one-way ANOVA with Tukey’s multiple comparisons test was used to determine the *P*-values between the different treatments.

### ROS accumulation assays

The ROS measurements were performed as previously described (62). Briefly, *N. benthamiana* leaves were infiltrated with the *A. citrulli* WT and mutant suspension at OD_600_ = 0.2 (10^8^ CFU/mL). The leaves infiltrated with 10 mM MgCl_2_ were taken as the negative control. At 15 hpi, 12 leaf disks (5 mm in diameter) were collected and placed into wells of a 96-well plate which was presupplied with 100 µL of sterile water. Then, 100 µL of L-012 (Wako, Japan) at 0.5 mM in 10 mM MOPS (morpholinepropanesulfonic acid)-KOH buffer (pH 7.4) was added. Chemiluminescence was recorded immediately using a Synergy H1 Hybrid Microplate Reader (BioTek Instruments, USA) for 16 h with a 15 min interval. This experiment was repeated three times. *P*-values were calculated by one-way ANOVA with Tukey’s multiple comparisons test.

### Sample preparation for RNA-seq

Overnight-grown bacterial cells of *A. citrulli* WT, ∆*tssM*, ∆*hrcC,* and ∆*hrcC*∆*tssM* were adjusted to OD_600_ = 0.2 (10^8^ CFU/mL) with 10 mM MgCl_2_. Different bacterial suspensions were infiltrated into cotyledons of 14-day-old watermelon (Jingxin No. 2) seedlings. After being infiltrated for 5 h, the leaves were harvested, frozen in liquid nitrogen, and stored at −80°C for RNA extraction. Total RNA was extracted using the TRNzol Universal total RNA extraction reagent (TIANGEN Biotech, DP424) according to the manufacturer’s instructions. The quality of total RNA was assessed by electrophoresis using 1% (w/v) agarose gel. There were three independent experiments in this assay.

### RNA-Seq data processing and analyses

The library preparation and transcriptome sequencing were performed by Novogene. In brief, RNA integrity was assessed using the RNA Nano 6000 Assay Kit of the Bioanalyzer 2100 system (Agilent Technologies, CA, USA). mRNA was purified using poly-T oligo-attached magnetic beads and fragmented by divalent cations under elevated temperature. The first-strand cDNA was synthesized using random hexamer primer and M-MuLV Reverse Transcriptase, followed by the degradation of the RNA strand by RNase H. The second strand cDNA synthesis was performed using DNA Polymerase I and RNase H. The purified double-stranded cDNA was connected to the sequencing adapter after adenylation of 3′ ends. AMPure XP system (Beckman Coulter, Beverly, USA) was used to select the cDNA in a length of 370–420 bp. After amplification of the library fragments, the PCR products were purified with the AMPure XP system, and library quality was assessed on the Agilent Bioanalyzer 2100 system. According to the manufacturer’s instructions, the index-coded samples were clustered on a cBot Cluster Generation System using TruSeq PE Cluster Kit v3-cBot-HS (Illumia). The sequencing was on an Illumina Novaseq platform, and 150 bp paired-end reads were generated.

Clean data were obtained by removing reads containing adapter, ploy-N and those reads with a phred ≤ 20 bases more than 50% of the total read length from raw data. Watermelon (97103) v2 Genome (http://cucurbitgenomics.org/organism/21) was used as the reference genome. Hisat2 v2.0.5 was used for the paired-end clean reads alignment to the reference genome. featureCounts (1.5.0-p3) was used to count the reads numbers mapped to each gene. FPKM (expected number of Fragments Per Kilobase of transcript sequence per Millions base pairs sequenced) of each gene was calculated based on the length of the gene and reads count mapped to this gene. Differential expression analysis was performed using the DESeq2 R package (1.20.0). The resulting *P*-values were adjusted using Benjamini and Hochberg’s approach for controlling the false discovery rate. DEGs were filtered at a cutoff of |Log_2_ fold change| > 1 and *P*-value < 0.05 by DESeq2. Gene ontology (GO) enrichment analyses and KEGG pathways enrichment of differential expression genes were implemented by the clusterProfiler R package (3.4.4).

### Quantitative real-time PCR analysis

Total RNA from WT, ∆*tssM*, ∆*hrcC*, and ∆*hrcC*∆*tssM* infiltrated watermelon cotyledons was extracted in the same way as for RNA-seq experiments using the TRNzol Universal total RNA extraction reagent (TIANGEN Biotech, DP424) according to the manufacturer’s instructions. Reverse-transcription PCR and quantitative real-time PCR (qRT-PCR) were performed using the PrimeScript RT reagent Kit with DNA Eraser (Takara, Dalian, China) and the MightyAmp for Real Time (SYBR Plus) (Takara), respectively. CFX Connect Real-Time PCR Detection System (BIO-RAD, #1855201) was used. *Actin* was used as the reference gene. A total of 19 genes involved in different pathways corresponding to RNA-seq data were selected to assess gene expression levels (Fig. S5). Primers for qRT-PCR were designed using Primer Premier 5.0 (63) and listed in Table S2. The 2^−ΔΔCt^ method (64) was used to calculate the expression level of the tested genes. This experiment was carried out three times with three replicates per treatment.

## Data Availability

The RNA-Seq data supporting the findings of this study are openly available at the NCBI Sequence Read Archive under the BioProject PRJNA846513.

## SUPPLEMENTAL MATERIAL

Figure S1 mbio.00459-23-s0001.docx Concentration of the *A. citrulli* when infiltrated to test the proliferation on watermelon.

Figure S2 mbio.00459-23-s0002.docx Phenotype of colonies of phyllosphere bacteria isolated from watermelon seedlings.

Figure S3 mbio.00459-23-s0003.docx Competition assay of *A. citrulli* (Ac) against watermelon phyllosphere bacteria.

Figure S4 mbio.00459-23-s0004.docx Summary of RNA-seq data.

Figure S5 mbio.00459-23-s0005.docx Assessment of gene expression by qRT-PCR of nineteen genes selected from the RNA-Seq data.

Figure S6 mbio.00459-23-s0006.docx KEGG pathway and GO analysis of comparison of ∆*tssM*_vs_WT.

Figure S7 mbio.00459-23-s0007.docx KEGG pathway and GO analysis of comparison of ∆*hrcC*_vs_WT.

Figure S8 mbio.00459-23-s0008.docx Effect of T6SS and T3SS on biofilm formation of *A. citrulli* AAC00-1.

TABLE S1 mbio.00459-23-s0009.xlsx Differentially expressed genes in the ∆*tssM* infiltrated sample compared to the WT infiltrated sample.

TABLE S2 mbio.00459-23-s0010.docx Strains, plasmids, and primers in this study.

ASM does not own the copyrights to Supplemental Material that may be linked to, or accessed through, an article. The authors have granted ASM a non-exclusive, world-wide license to publish the Supplemental Material files. Please contact the corresponding author directly for reuse.
