# Supplementary material for: Differential plant cell responses to Acidovorax citrulli T3SS and T6SS reveal an effective strategy for controlling plant-associated pathogens
Source: mBio. 2023 Jun 8;14(4):e00459-23. doi: 10.1128/mbio.00459-23 (PMC10470598; doi:10.1128/mbio.00459-23)
Supplement: Figure S1 — Concentration of the A. citrulli when infiltrated to test the proliferation on watermelon. [file mbio.00459-23-s0001.docx]

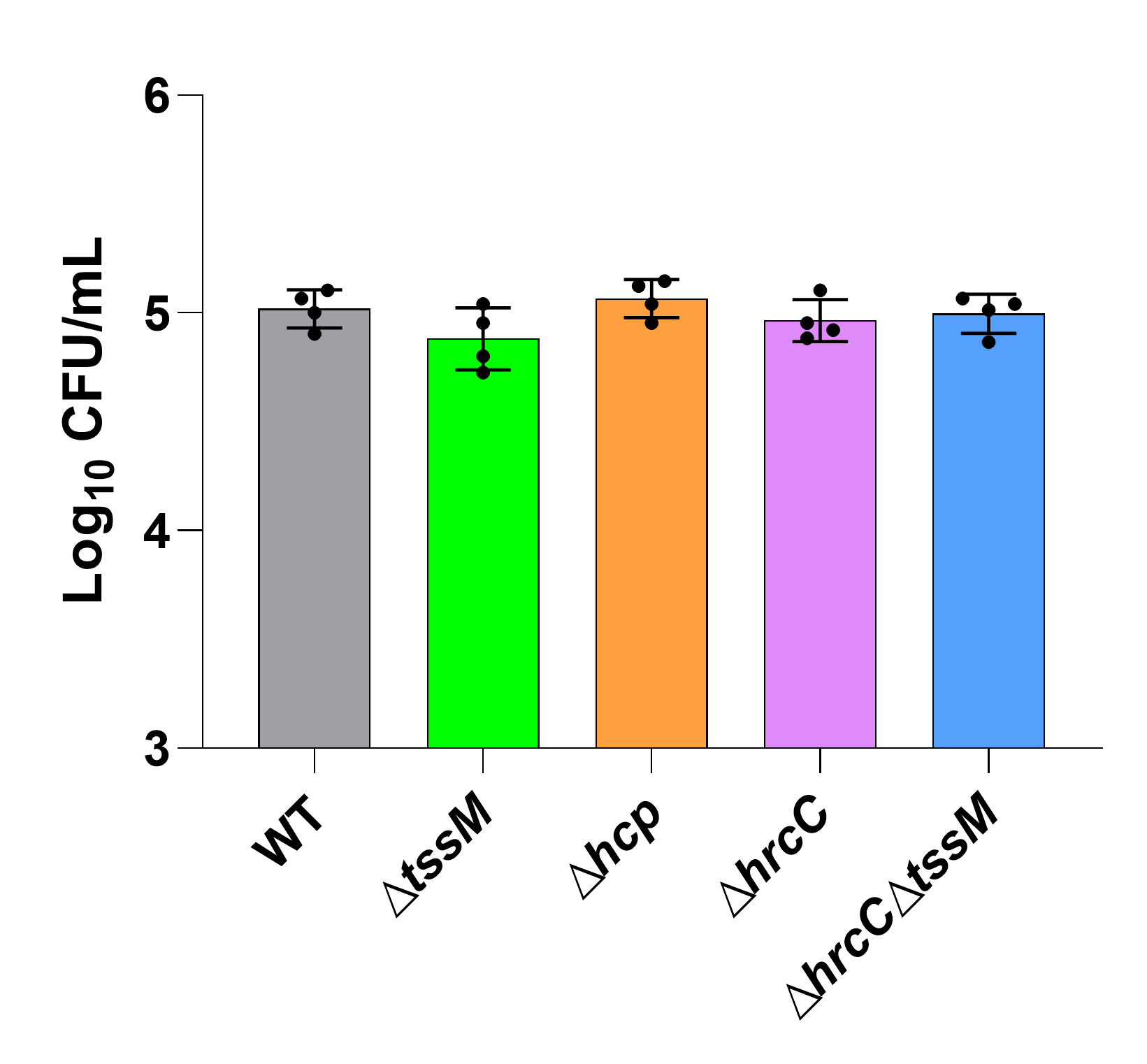


**FIG S1** Concentration of the *A. citrulli* when infiltrated to test the proliferation on watermelon. The error bars represent the standard deviation of the means from two independent experiments, and each was counted on two LB plates with kanamycin. WT, *A. citrulli* AAC00-1 wild type; ∆*tssM* and ∆*hcp*, T6SS-null strains; ∆*hrcC*, T3SS-null strain; ∆*hrcC*∆*tssM*, mutant that both T3SS and T6SS are inactive. Statistical significance was calculated by one-way ANOVA with Tukey’s multiple comparisons test and there are no significant differences between each sample and the others.
