# Supplementary material for: Differential plant cell responses to Acidovorax citrulli T3SS and T6SS reveal an effective strategy for controlling plant-associated pathogens
Source: mBio. 2023 Jun 8;14(4):e00459-23. doi: 10.1128/mbio.00459-23 (PMC10470598; doi:10.1128/mbio.00459-23)
Supplement: Figure S2 — Phenotype of colonies of phyllosphere bacteria isolated from watermelon seedlings. [file mbio.00459-23-s0002.docx]

**
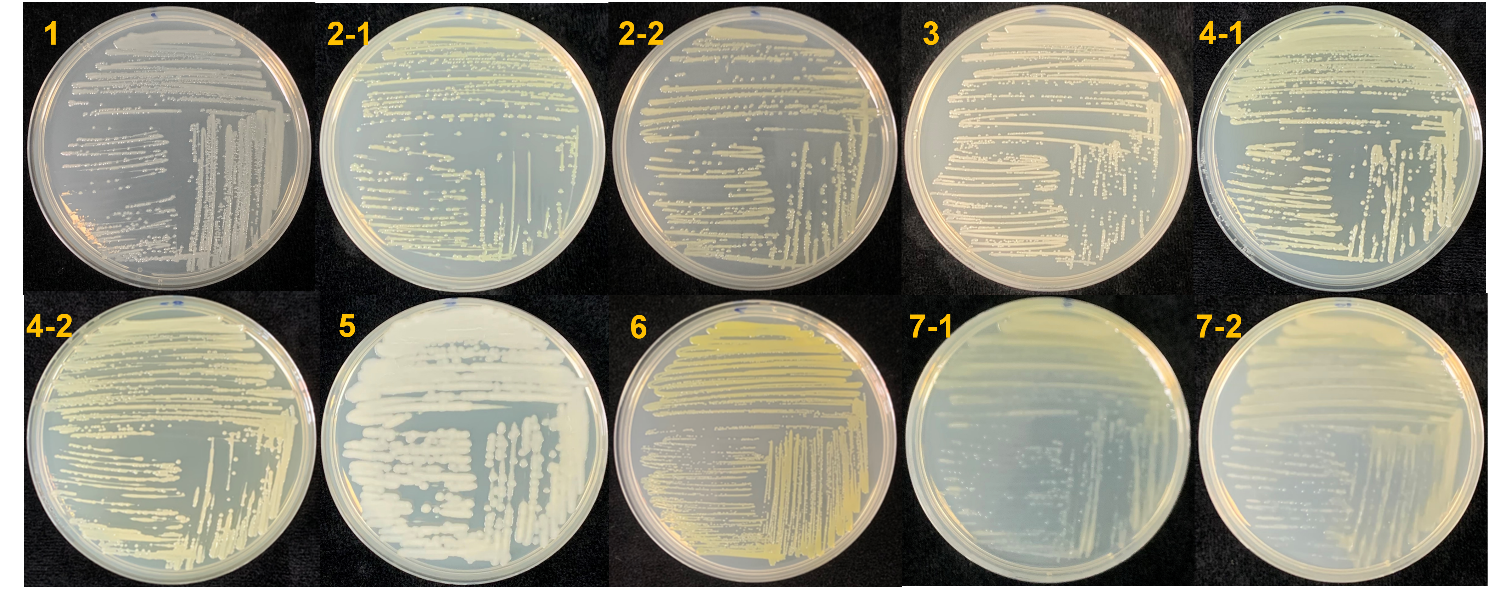
**

**FIG S2** Phenotype of colonies of phyllosphere bacteria isolated from watermelon seedlings. Watermelon cotyledons and true leaves were frozen in liquid nitrogen and ground into powder at 45 Hz for 100 s with the Tissue Lyser Wonbio-L. One milliliter of 0.85% NaCl was added and mixed well. The homogenate was streaked on LB plate and incubated at 28 °C for 48 h. These ten strains were photographed and identified according to the sequence of their 16S rDNA.
