# Supplementary material for: Differential plant cell responses to Acidovorax citrulli T3SS and T6SS reveal an effective strategy for controlling plant-associated pathogens
Source: mBio. 2023 Jun 8;14(4):e00459-23. doi: 10.1128/mbio.00459-23 (PMC10470598; doi:10.1128/mbio.00459-23)
Supplement: Figure S3 — Competition assay of A. citrulli (Ac) against watermelon phyllosphere bacteria. [file mbio.00459-23-s0003.docx]

**
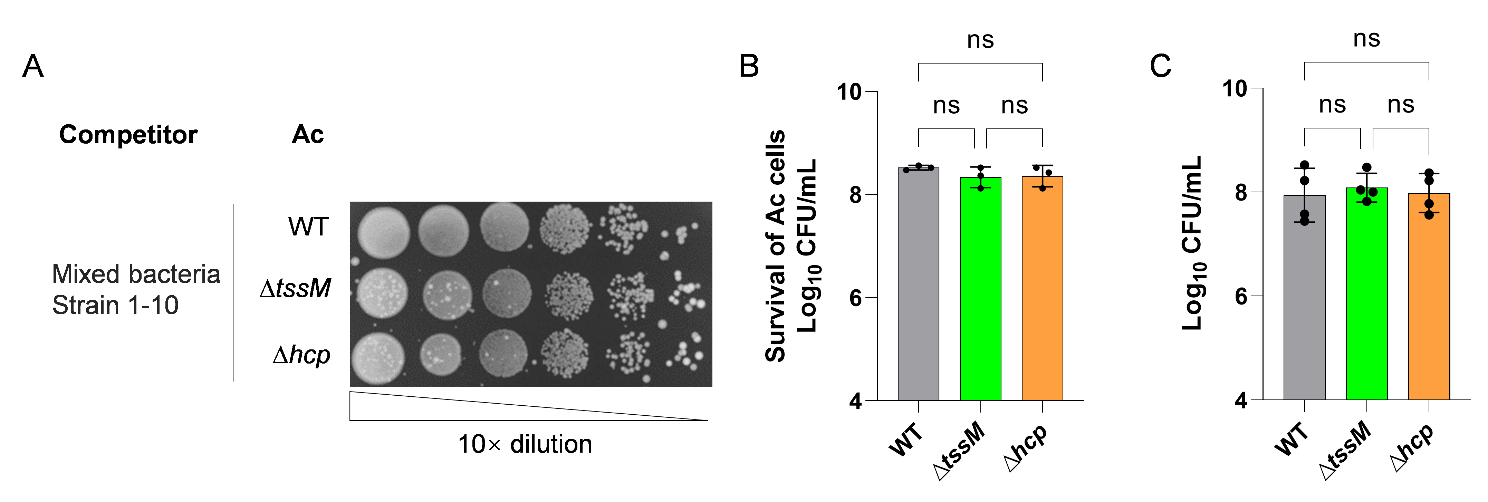
**

**FIG S3** Competition assay of *A*. *citrulli* (Ac) against watermelon phyllosphere bacteria. (A) Appearance of survival of *A. citrulli* strains after co-incubated with the phyllosphere bacteria on LB plate with kanamycin for 6 h. (B) Survival of *A. citrulli* strains after co-incubated with the phyllosphere bacteria for 24 h *in vitro* (Ac:Competitor_mix_=OD10:10). The error bars represent standard deviation of the means from three independent experiments. (C) Concentration of *A. citrulli* (Ac) for co-infiltrated with watermelon phyllosphere bacteria on watermelon cotyledons (Ac:Competitor_mix_ =OD1:10). The error bars represent standard deviation of the means from two independent experiments, each was counted on two LB plates. WT, *A. citrulli* AAC00-1 wild type; ∆*tssM* and ∆*hcp*, T6SS-null strains. Statistical significance was calculated by one-way ANOVA with Tukey’s multiple comparisons test. ns, no significant difference.
