# Supplementary material for: Differential plant cell responses to Acidovorax citrulli T3SS and T6SS reveal an effective strategy for controlling plant-associated pathogens
Source: mBio. 2023 Jun 8;14(4):e00459-23. doi: 10.1128/mbio.00459-23 (PMC10470598; doi:10.1128/mbio.00459-23)
Supplement: Figure S4 — Summary of RNA-seq data. [file mbio.00459-23-s0004.docx]

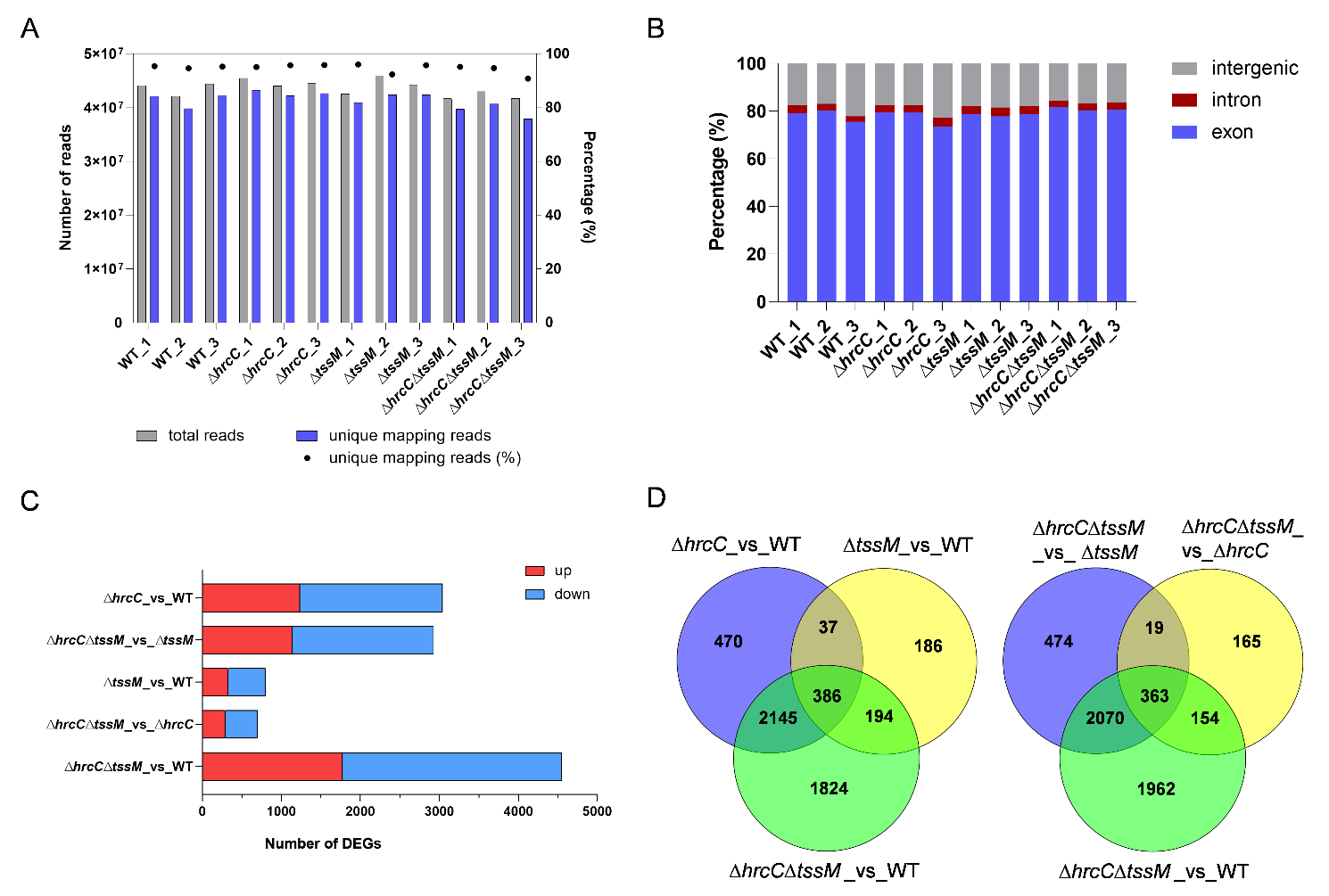


**FIG S4** Summary of RNA-seq data. (A) Number of the total reads, unique mapping reads and percentage of unique mapping reads in different treatments. (B) Percentage of reads mapping to the intergenic, intron and exon of the watermelon genome in different treatments. (C) Number of identified genes triggered by T3SS and T6SS. (D) Venn diagram representing the overlap of differentially expressed genes identified in different comparison groups. WT, *A. citrulli* AAC00-1 wild type; ∆*tssM*, T6SS-null strain; ∆*hrcC*, T3SS-null strain; ∆*hrcC*∆*tssM*, mutant that both T3SS and T6SS are inactive. Watermelon (97103) v2 Genome (<http://cucurbitgenomics.org/organism/21>) was used as reference genome.
