# Supplementary material for: Differential plant cell responses to Acidovorax citrulli T3SS and T6SS reveal an effective strategy for controlling plant-associated pathogens
Source: mBio. 2023 Jun 8;14(4):e00459-23. doi: 10.1128/mbio.00459-23 (PMC10470598; doi:10.1128/mbio.00459-23)
Supplement: Figure S5 — Assessment of gene expression by qRT-PCR of nineteen genes selected from the RNA-Seq data. [file mbio.00459-23-s0005.docx]

**
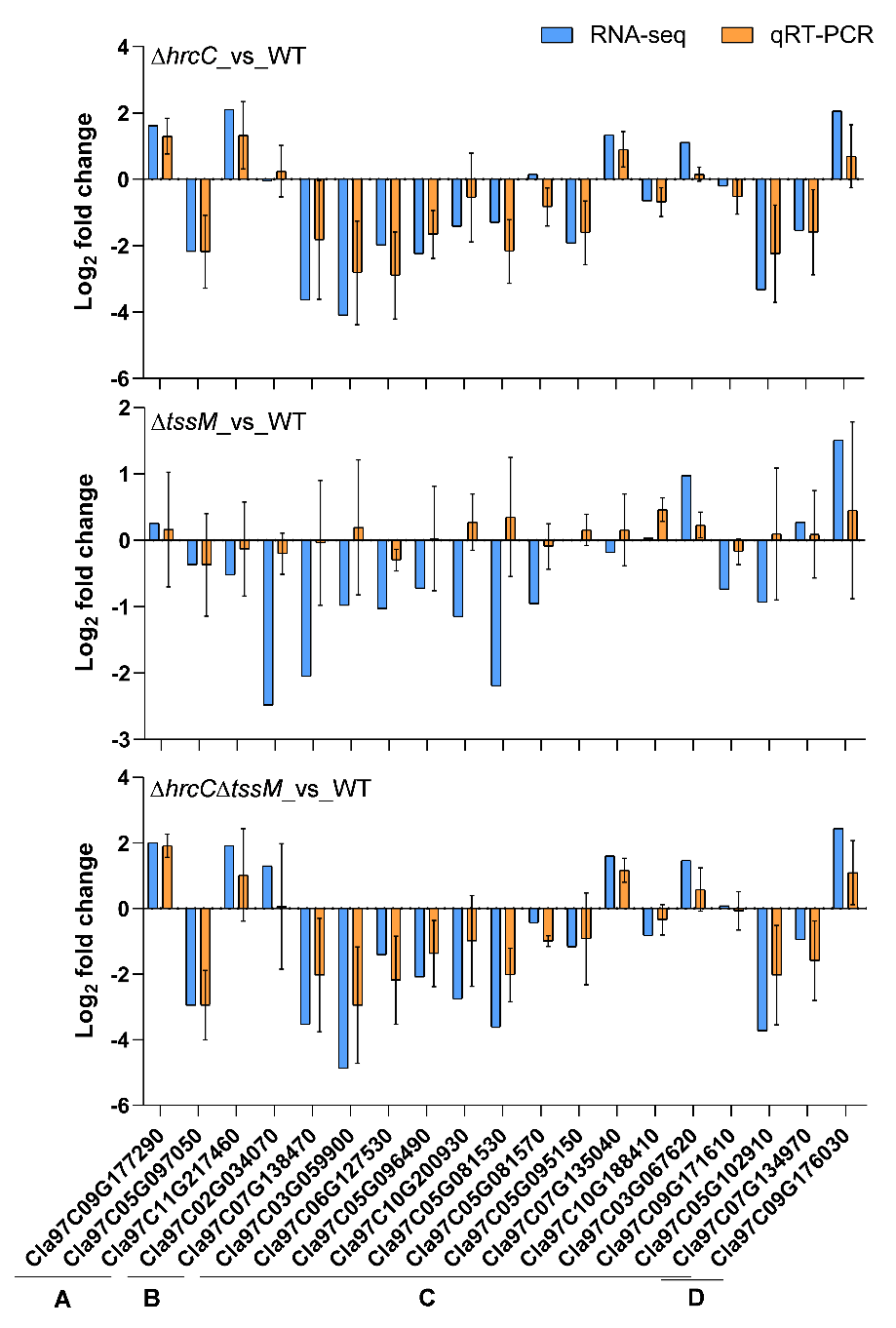
**

**FIG S5** Assessment of gene expression by qRT-PCR of nineteen genes selected from the RNA-Seq data. The error bars represent standard deviation of the means from three independent experiments, each containing three replicates per gene. WT, *A. citrulli* AAC00-1 wild type; ∆*tssM*, T6SS-null strain; ∆*hrcC*, T3SS-null strain; ∆*hrcC*∆*tssM*, mutant that both T3SS and T6SS are inactive. Letters mean different pathways: A, phenylpropanoid biosynthesis; B, plant hormone signal transduction; C, plant-pathogen interaction; D, MAPK signaling pathway-plant.
