# Supplementary material for: Differential plant cell responses to Acidovorax citrulli T3SS and T6SS reveal an effective strategy for controlling plant-associated pathogens
Source: mBio. 2023 Jun 8;14(4):e00459-23. doi: 10.1128/mbio.00459-23 (PMC10470598; doi:10.1128/mbio.00459-23)
Supplement: Figure S6 — KEGG pathway and GO analysis of comparison of ∆tssM_vs_WT. [file mbio.00459-23-s0006.docx]

**
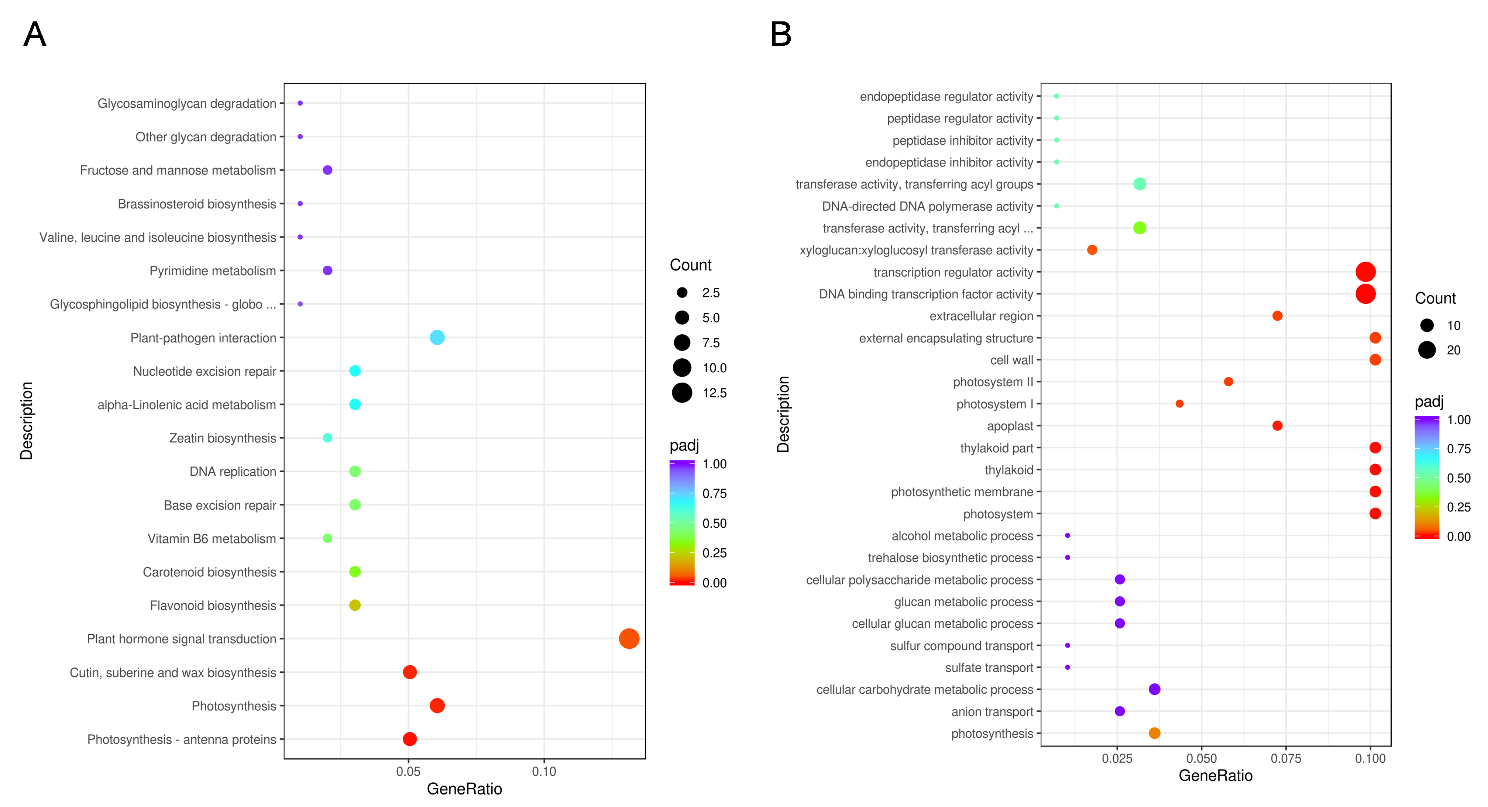
FIG S6** KEGG (Kyoto Encyclopedia of Genes and Genomes) pathway and GO (Gene Ontology) analysis of comparison of ∆*tssM*_vs_WT. (A) KEGG. (B) GO. Differentially expressed genes were filtered at a cutoff of |Log_2_ fold change|>1 and adjusted *P*-value <0.05. WT, *A. citrulli* AAC00-1 wild type; ∆*tssM*, T6SS-null strain.
