# Supplementary material for: Differential plant cell responses to Acidovorax citrulli T3SS and T6SS reveal an effective strategy for controlling plant-associated pathogens
Source: mBio. 2023 Jun 8;14(4):e00459-23. doi: 10.1128/mbio.00459-23 (PMC10470598; doi:10.1128/mbio.00459-23)
Supplement: Figure S8 — Effect of T6SS and T3SS on biofilm formation of A. citrulli AAC00-1. [file mbio.00459-23-s0008.docx]

**
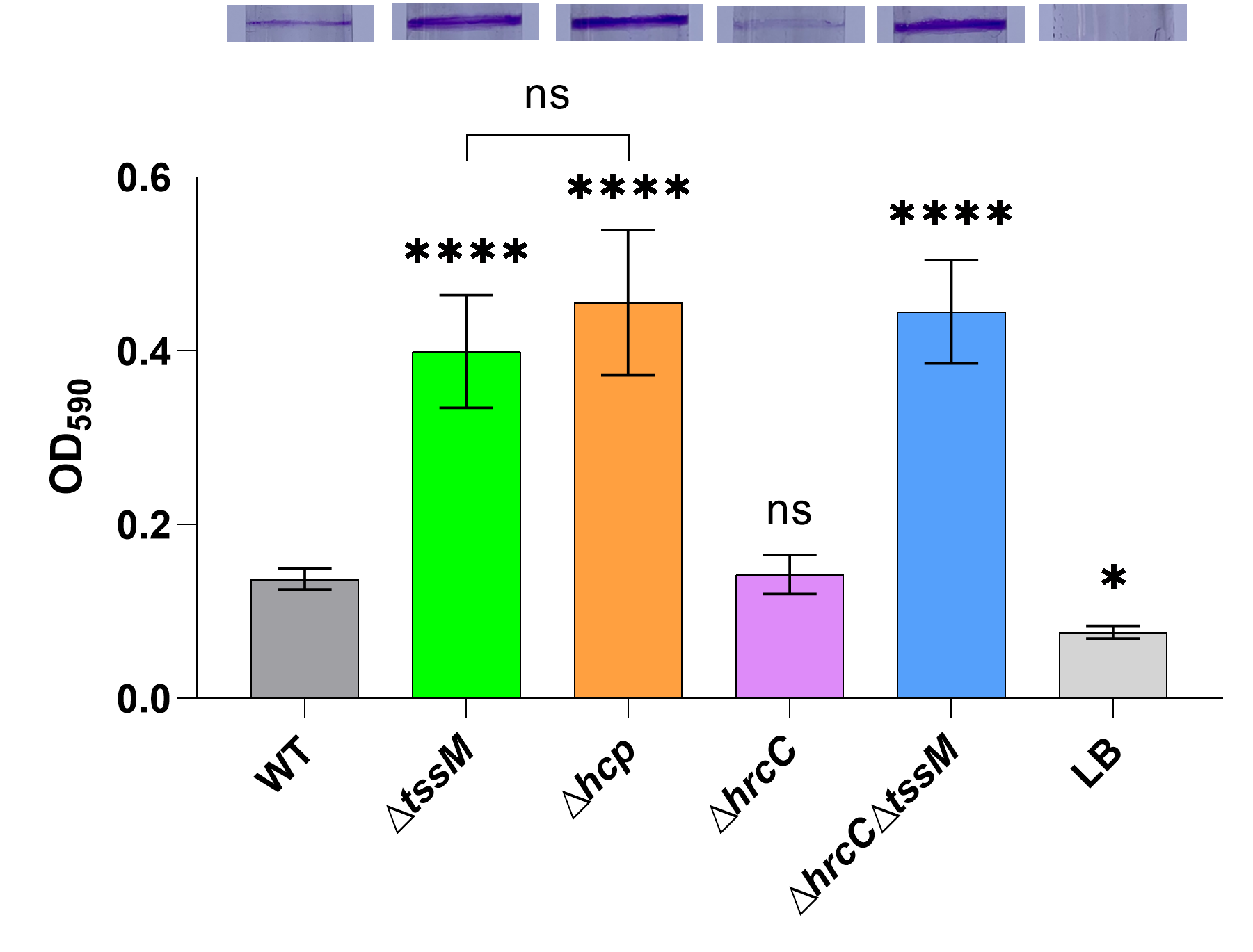
**

**FIG S8** Effect of T6SS and T3SS on biofilm formation of *A. citrulli* AAC00-1. Biofilm of different strains formed in glass tubes after staining with crystal violet, and stained biofilm solubilized with ethanol and measured the optical density at 590 nm. WT, *A. citrulli* AAC00-1 wild type; ∆*tssM* and ∆*hcp*, T6SS-null strains; ∆*hrcC*, T3SS-null strain; ∆*hrcC*∆*tssM*, mutant that both T3SS and T6SS are inactive. The error bars represent the standard deviation of the means from three independent experiments, each containing four replicates per treatment. Statistical significance was calculated by one-way ANOVA with Tukey’s multiple comparisons test. **** *P* <0.0001, * *P* <0.05; ns, not significant.
