# Supplementary material for: Differential plant cell responses to Acidovorax citrulli T3SS and T6SS reveal an effective strategy for controlling plant-associated pathogens
Source: mBio. 2023 Jun 8;14(4):e00459-23. doi: 10.1128/mbio.00459-23 (PMC10470598; doi:10.1128/mbio.00459-23)
Supplement: TABLE S2 — Strains, plasmids, and primers in this study. [file mbio.00459-23-s0010.docx]

**Table S2. Strains, plasmids and primers in this study**

| **Strain** | **Genotype** | **Description** | **Reference** |
| --- | --- | --- | --- |
| *Acidovorax citrulli* AAC00-1 | Parental | Parental strain | Lab stock |
|  | Δ*tssM* | T6SS null, in-frame deletion of *tssM* | ^[1]^ |
|  | Δ*hcp* | T6SS null, in-frame deletion of *hcp* | This study |
|  | Δ*hrcC* | T3SS null, in-frame deletion of *hrcC* | This study |
|  | Δ*hrcC*Δ*tssM* | T3SS and T6SS null, in-frame deletion of *hrcC* and *tssM* | This study |
|  | Parental | Parental strain with the pBBR1MCS-2 plasmid, Km^+^ | This study |
|  | Δ*tssM* | Δ*tssM* strain with the pBBR1MCS-2 plasmid, Km^+^ | This study |
|  | Δ*hcp* | Δ*hcp* strain with the pBBR1MCS-2 plasmid, Km^+^ | This study |
|  | Parental | Parental strain with the pBBR1MCS-5 plasmid, Gm^+^ | This study |
|  | Δ*tssM* | Δ*tssM* strain with the pBBR1MCS-5 plasmid, Gm^+^ | This study |
|  | Δ*hcp* | Δ*hcp* strain with the pBBR1MCS-5 plasmid, Gm^+^ | This study |
|  | Δ*hrcC*Δ*tssM* | Δ*hrcC*Δ*tssM* strain with the pBBR1MCS-5 plasmid, Gm^+^ | This study |
| *E. coli* |  |  |  |
| T-Fast |  | Strain used for cloning and gene expression | TIANGEN |
| WM6026 |  | Strain used for conjugation | Lab stock |
| DAEC 2787 |  | Natural resistance to streptomycin, Str^+^ | Lab stock |
| ETEC H-10407 |  | Natural resistance to streptomycin, Str^+^ | Lab stock |
| *Citrobacter rodentium* |  | Wild type strain with the pBAD24 plasmid, Km^+^ | Lab stock |
| *Klebsiella pneumoniae* |  | Wild type strain with the pET28a plasmid, Km^+^ | Lab stock |
| *Photorhabdus asymbiotica* |  | Natural resistance to streptomycin, Str^+^ | Lab stock |
| *Vibrio cholerae* C6706 |  | Natural resistance to streptomycin, Str^+^ | Lab stock |
| *V*. *parahaemolyticus* |  | Natural resistance to streptomycin, Str^+^ | Lab stock |
| *Pseudomonas syringae* pv. *syringae* |  | Natural resistance to streptomycin, Str^+^ | Lab stock |
| *P. syringae* pv. tomato PtoT1 |  | Wild type strain with the pBBR1MCS-2 plasmid, Km^+^ | Lab stock |
| *X. oryzae* pv*.oryzae* PXO99A |  | Wild type strain with the pBBR1MCS-2 plasmid, Km^+^ | Lab stock |
| **Plasmid** | **Description** | | **Reference** |
| pEXG2.0 | Suicidal conjugation vector for chromosomal allelic changes | | Lab stock |
| pEXG2.0-Ac-hcp | Suicidal vector to construct in-frame deletion mutant of *hcp* (Aave_1465) | | This study |
| pK18mobsacB | Suicidal conjugation vector for chromosomal allelic changes | | Lab stock |
| pK18-Ac-hrcC | Suicidal vector to construct in-frame deletion mutant of *hrcC* (Aave_0474) | | This study |
| pBBR1MCS-2 | A broad-host-range cloning vector, kanamycin resistance | | Lab stock |

**Supplementary References**

1. Pei TT, Kan Y, Wang ZH, Tang MX, Li H, Yan S, Cui Y, Zheng HY, Luo H, Liang X, Dong T. 2022. Delivery of an Rhs-family nuclease effector reveals direct penetration of the gram-positive cell envelope by a type VI secretion system in *Acidovorax citrulli*. mLife 1:66-78.

| **Primer name** | **Sequence (forward/reverse)** | **Length (bp)** | **Description** |
| --- | --- | --- | --- |
| KO_*hcp*-1 | TACCGAATTCGAGCTCGAGCCCGGGAAGCACCGTCTTGACCGTATG | 976 | For deleting the *hcp* gene, located upstream of the *hcp* gene |
| KO_*hcp*-2 | TTGAAGGTGCCAGCGGCGAAATCAAGGAAAACAAAGAAGCCTGA |  |  |
| KO_*hcp*-3 | GCTTCTTTGTTTTCCTTGATTTCGCCGCTGGCACCTT | 824 | For deleting the *hcp* gene, located downstream of the *hcp* gene |
| KO_*hcp*-4 | CTGCAGGTCGACTCTGAGATCTGGGACCGCACATGCACCT |  |  |
| KO_*hcp*-5 | CTTGGGCATGCGAGGCT | 1902 | For confirming the deletion of the *hcp* mutant |
| KO_*hcp*-6 | CGGATGGGAAAACTTGGCC |  |  |
| KO_*hrcC*-1 | GATTACGAATTCGAGCTCGGGTGAGCGAAAGGTGCTTCAC | 967 | For deleting the *hrcC* gene, located upstream of the *hrcC* gene |
| KO_*hrcC*-2 | GCCCAAAATTTCCGCGAGGTTCGCAAGCCATGA |  |  |
| KO_*hrcC*-3 | TGGCTTGCGAACCTCGCGGAAATTTTGGGCATC | 1005 | For deleting the *hrcC* gene, located downstream of the *hrcC* gene |
| KO_*hrcC*-4 | AGTCACGACGTTGTAAAACGATATTCGTGCAGCGCGTAGTT |  |  |
| KO_*hrcC*-5 | GTCGTGCCTTCCTCAGACCG | 2800 | For confirming the deletion of the *hrcC* mutant |
| KO_*hrcC*-6 | CAGCAGCAGCAGCCCTTCA |  |  |
| *Actin*-F | CCATGTATGTTGCCATCCAG | 135 | For testing the transcriptional level of reference gene *Actin* |
| *Actin*-R | GGATAGCATGGGGTAGAGCA |  |  |
| Cla97C02G034070-F | TTGGTGTCGGGCCAGTCT | 146 | For testing the transcriptional level of Cla97C02G034070 |
| Cla97C02G034070-R | CGCCGTCATCTCGTCATAC |  |  |
| Cla97C03G059900-F | TGACGAAGTGCAGCGGATGA | 86 | For testing the transcriptional level of Cla97C03G059900 |
| Cla97C03G059900-R | CGCGGGCGAAATCTGTGA |  |  |
| Cla97C03G067620-F | GCCTACCTTGTGGCTGAA | 98 | For testing the transcriptional level of Cla97C03G067620 |
| Cla97C03G067620-R | GTGACTGTGAACGAACCC |  |  |
| Cla97C05G081530-F | TTGCGAGTCATCGGAAAC | 117 | For testing the transcriptional level of Cla97C05G081530 |
| Cla97C05G081530-R | GCACTCCTCGTCTGAAACT |  |  |
| Cla97C05G081570-F | TGACCCATCACAAGCAAC | 147 | For testing the transcriptional level of Cla97C05G081570 |
| Cla97C05G081570-R | CCCAAATATCCGAAGACA |  |  |
| Cla97C05G095150-F | ACGACTGTCGGCGTATCAT | 188 | For testing the transcriptional level of Cla97C05G095150 |
| Cla97C05G095150-R | TCCTCACCTTCTTCTCCC |  |  |
| Cla97C05G096490-F | CCTTCTCCTATGGCTCAG | 96 | For testing the transcriptional level of Cla97C05G096490 |
| Cla97C05G096490-R | TGTAAGAGGGAATAGACTGC |  |  |
| Cla97C05G097050-F | TCCGGTTGCTCTTCCATG | 107 | For testing the transcriptional level of Cla97C05G097050 |
| Cla97C05G097050-R | TCCTCCAAGCGACCTGTT |  |  |
| Cla97C05G102910-F | GTCGGAGGAGGAGTTTGAG | 169 | For testing the transcriptional level of Cla97C05G102910 |
| Cla97C05G102910-R | CTGCATCTTGACTTCGGTAA |  |  |
| Cla97C06G127530-F | TACGATACCGAAGGTTGTG | 111 | For testing the transcriptional level of Cla97C06G127530 |
| Cla97C06G127530-R | GAAGCGACGAATCATCAA |  |  |
| Cla97C07G134970-F | ATTCTAAATGACCACGACTC | 133 | For testing the transcriptional level of Cla97C07G134970 |
| Cla97C07G134970-R | CGTCACCTCCACAAACTC |  |  |
| Cla97C07G135040-F | TTCTGCCTCCATTTCTGA | 148 | For testing the transcriptional level of Cla97C07G135040 |
| Cla97C07G135040-R | GCATACGCTATCCGCTTT |  |  |
| Cla97C07G138470-F | TTTAGTCGCCAAAGAAGC | 122 | For testing the transcriptional level of Cla97C07G138470 |
| Cla97C07G138470-R | ATCGGAATAGTGAAATCAGAAC |  |  |
| Cla97C09G171610-F | GGAAAGCGAAGGGAATGT | 149 | For testing the transcriptional level of Cla97C09G171610 |
| Cla97C09G171610-R | TGGATTTGGTTGGAGCAG |  |  |
| Cla97C09G176030-F | TTGAACCTTTCGGCAAAC | 107 | For testing the transcriptional level of Cla97C09G176030 |
| Cla97C09G176030-R | ACCGGACCCAAGAAGTAA |  |  |
| Cla97C09G177290-F | TTTTGTCCAACGCTTTCT | 128 | For testing the transcriptional level of Cla97C09G177290 |
| Cla97C09G177290-R | CTTTCCGAATTTCTCCTT |  |  |
| Cla97C10G188410-F | GTCCAGTAACGGGTCAAA | 162 | For testing the transcriptional level of Cla97C10G188410 |
| Cla97C10G188410-R | GTCCAGGTTGGTTCATTC |  |  |
| Cla97C10G200930-F | GTGGTTGGTAGCCCTTAT | 143 | For testing the transcriptional level of Cla97C10G200930 |
| Cla97C10G200930-R | ATTCCTTGCTCCGTCTCA |  |  |
| Cla97C11G217460-F | TATGGTGTTTACCGTTTATG | 182 | For testing the transcriptional level of Cla97C11G217460 |
| Cla97C11G217460-R | GCTTTCTAATGACTATCCCT |  |  |
